# Supplementary material for: A dual-selective thermal emitter with enhanced subambient radiative cooling performance
Source: Nat Commun. 2024 Jan 27;15:815. doi: 10.1038/s41467-024-45095-4 (PMC10821923; doi:10.1038/s41467-024-45095-4)
Supplement: Supplementary file 1 — Supplementary Information [file 41467_2024_45095_MOESM1_ESM.pdf]

Supplementary Information for

A Dual-Selective Thermal Emitter with Enhanced Subambient Radiative Cooling Performance

Xueke Wu[1], Jinlei Li[2], Fei Xie[3], Xun-En Wu[4], Siming Zhao[1], Qinyuan Jiang[1], Shiliang Zhang[1], Baoshun Wang[1], Yunrui Li[1], Di Gao[1], Run Li[1], Fei Wang[1], Ya Huang[1], Yanlong Zhao[1], Yingying Zhang[4], Wei Li[3], Jia Zhu[2], Rufan Zhang[1*]

[1]Beijing Key Laboratory of Green Chemical Reaction Engineering and Technology, Department of Chemical Engineering, Tsinghua University, Beijing 100084, China.

[2]National Laboratory of Solid State Microstructures, College of Engineering and Applied Sciences, Jiangsu Key Laboratory of Artificial Functional Materials, Collaborative Innovation Center of Advanced Microstructures, Nanjing University, Nanjing 210023, China.

[3]GPL Photonics Laboratory, State Key Laboratory of Luminescence and Applications, Changchun Institute of Optics, Fine Mechanics and Physics, Chinese Academy of Sciences, Changchun, Jilin, 130033 P. R. China.

[4]Key Laboratory of Organic Optoelectronics and Molecular Engineering of the Ministry of Education, Department of Chemistry, Tsinghua University, Beijing 100084, PR China.

*Email: zhangrufan@tsinghua.edu.cn

Supplementary Text

Supplementary text 1. Modelling of the radiative cooling performance of a dual-selective thermal emitter

The dual-selective RC model is featured with a highly selective absorption/emission only within the two atmospheric windows (8–13 μm and 16–25 μm, respectively) and a high reflectance in the non-window MIR wavebands (bottom of Fig. 1a and Fig. 1b). To evaluate the cooling potential of the dual-selective RC model, The atmospheric transparency data in an arid climate (to maximize the cooling effect of the second window) were used for the calculation. The typical theoretical models of non-selective and mono-selective emitters (Fig. 1b) were employed for comparison. The former is featured with a high absorption/emission over the entire MIR broadband (Fig. 1a (top) and 1b), and the latter is featured with a high absorption/emission only in the first atmospheric window of 8–13 μm waveband and a high reflectance in the remaining MIR wavebands (Fig. 1a (middle) and 1b).

Based on a simple one-dimensional heat transfer model[1], the energy balance equation for the thermal emitters combined with radiative and non-radiative thermal transfer is

$$P_{\mathrm{net}} = P_{\mathrm{rad}} - P_{\mathrm{atm}} - P_{\mathrm{sun}} - P_{\mathrm{non\text{-}rad}} \quad (1)$$

, where P_{net} is the net cooling power of the thermal emitter; P_{rad} is the emitted thermal radiation from the thermal emitter; P_{atm} is the absorbed thermal radiation from the atmosphere; P_{sun} is the absorbed thermal radiation from sunlight; $P_{\mathrm{non\text{-}rad}}$ is the non-radiative heat transfer by thermal conduction and thermal convention.

The P_{rad} and P_{atm} are described by Fourier's law, which give the equations

$$P_{\text{rad}} = 2\pi \int_0^{\pi/2} sin\theta cos\theta d\theta \int_0^{\infty} \varepsilon I_{bb}(T_{\text{sample}}) d\lambda \quad (2)$$

$$P_{\text{atm}} = 2\pi \int_0^{\pi/2} sin\theta cos\theta d\theta \int_0^{\infty} \varepsilon_{atm} I_{bb}(T_{\text{ambient}}) d\lambda \quad (3)$$

, where $T_{\text{ambient}}$and T_{sample} are the temperature of the ambient air and the thermal emitter, respectively. ε_{atm} and ε are the emittance of the ambient air and the thermal emitter, respectively. λ is wavelength. And I_{bb} (T) is the blackbody radiation intensity. The I_{bb} (T) can be canculated from

$$I_{bb}\ (T) = \frac{4\pi c^2 \hbar}{\lambda^5} \frac{1}{e^{\frac{2\pi\hbar c}{\lambda k_B T}} - 1} \quad (4)$$

, where c, $\hbar$, and k_B are the speed of light (3×10^8 m s^{-1}), the reduced Planck constant (1.055×10^{-34} J s), and Boltzmann constant (1.381×10^{-23} J K^{-1}), respectively.

The non-radiative heat transfer is described by Fourier's law, which gives the equation

$$P_{\text{non-rad}} = h\,(T_{\text{ambient}} - T_{\text{sample}}) \quad (5)$$

, where h is the non-radiative heat transfer coefficient.

Base on the Eqns. 1–5, P_{net} of the three models (P_{sun} = 0 W m^{-2} for theoretical limit) can be calculated by the following equations.

For the non-selective RC model (ε = 1 in the entire MIR waveband of 4–25 μm):

$$P_{\text{net}}(\text{non-selective}) = 2\pi \int_0^{\pi/2} sin\theta cos\theta d\theta \int_4^{25} I_B(T_{\text{sample}}) d\lambda - 2\pi \int_0^{\pi/2} sin\theta cos\theta d\theta \int_4^{25} \varepsilon_{atm} I_B(T_{\text{ambient}}) d\lambda - h(T_{\text{ambient}} - T_{\text{sample}}) \quad (6)$$

For the mono-selective RC model (ε = 1 in the first atmospheric window of 8–13 μm waveband and ε = 0 in the remaining MIR waveband):

$$P_{\text{net}}(\text{mono-selective}) = 2\pi \int_0^{\pi/2} sin\theta cos\theta d\theta \int_8^{13} I_B(T_{\text{sample}}) d\lambda - 2\pi \int_0^{\pi/2} sin\theta cos\theta d\theta \int_8^{13} \varepsilon_{atm} I_B(T_{\text{ambient}}) d\lambda - h(T_{\text{ambient}} - T_{\text{sample}}) \quad (7)$$

For the dual-selective RC model (ε = 1 in the two atmospheric windows of 8–13 μm and 16–25 μm wavebands and ε = 0 in the non-window MIR wavebands):

$$P_{\text{net}}(\text{dual-selective}) = 2\pi \int_0^{\pi/2} sin\theta cos\theta d\theta [\int_8^{13} I_B(T_{\text{sample}}) d\lambda + \int_{16}^{25} I_B(T_{\text{sample}}) d\lambda] - 2\pi \int_0^{\pi/2} sin\theta cos\theta d\theta [\int_8^{13} \varepsilon_{atm} I_B(T_{\text{ambient}}) d\lambda + \int_{16}^{25} \varepsilon_{atm} I_B(T_{\text{ambient}}) d\lambda] - h(T_{\text{ambient}} - T_{\text{sample}}) \quad (8)$$

The ε_{atm} is described by

$$\varepsilon_{atm} = 1 - t(\lambda)^{1/cos\theta} \quad (9)$$

, where $t(\lambda)$ is the atmospheric transparency obtained from the ATRAN modelling software with a water vapor column of 1.0 mm and zero zenith angle[2,3].

The theoretical P_{net} of these three different RC models as a function of T_{sample} at a T_{ambient} of 20 °C was obtained when using different h values (0–8 W m^{-2} K^{-1}) (Fig. 1c and Supplementary Fig. 2), by which we can also obtain the theoretical limits of the subambient temperature reduction of the three models (as the temperature reduction of the thermal radiation reached a steady state when P_{net} = 0 W m^{-2}) (Fig. 1e and Supplementary Fig. 3).

Furthermore, the theoretical limits of P_{net} of these three different RC models as a function of $T_{ambient}$ were obtained when $T_{ambient}$ = T_{sample} (Fig. 1d and Supplementary Fig. 4), as the non-radiative heat can be excluded ($P_{non\text{-}rad}$ = $h\,(T_{ambient} - T_{sample})$ = 0).

Supplementary text 2. Relationship between the net cooling power and the subambient temperature reduction for dual-selective, mono-selective, and non-selective thermal emitters.

The radiative cooling performance of a sample can be expressed in two main ways: the radiative cooling power and the cooling temperature (temperature reduction or temperature difference), which are not positively correlated. The reason for this is that the former represents the rate of cooling (or heat dissipation), which depends on the "speed" at which heat is transferred from the Earth to the cold outer space. The latter is the end result of the heat transfer to the outer space and depends on the total amount of heat transferred to the outer space. Obviously, high cooling power does not imply large temperature reduction, and our ultimate goal in developing radiative cooling technology is to achieve high temperature reduction in most practical applications. Therefore, we prefer to use the subambient temperature reduction rather than the net cooling power to evaluate the radiative cooling performance of thermal emitters. The following is a detailed analysis of the radiative cooling performance (both net cooling power and subambient cooling temperature) for the three typical thermal emitters.

Non-selective thermal emitters can dissipate heat to the outer space across the entire mid-infrared waveband (note that non-window wavebands can also dissipate heat, even if the vast majority of the heat is absorbed/blocked by the atmosphere), whereas mono-selective thermal emitters can only dissipate heat through the narrow atmospheric transparent window of 8–13 μm. Therefore, non-selective thermal emitters have a much faster cooling rate (*i.e.,* higher net cooling power) than mono-selective thermal emitters. As for dual-selective thermal emitters, they can emit heat through both atmospheric transparent windows (8–13 μm and 16–25 μm), much faster than mono-selective thermal emitters and close to non-selective ones. Therefore, dual-selective thermal

emitters have a much higher net cooling power than mono-selective and close to non-selective thermal emitters (Fig. 1c).

As analyzed above, while non-selective thermal emitters dissipate heat quickly (with high cooling power), the trade-off is that most of the heat in the non-window band is absorbed/blocked by the atmosphere and returns to re-heat the emitters (*i.e.*, atmospheric parasitic heat). As a result, in ideal environments (h = 0 W m^{-2} K^{-1}), non-selective emitters emit the least total amount of heat to the outer space compared to the other two types of thermal emitters, resulting in the smallest temperature reduction ultimately achieved. In contrast, selective thermal emitters can dissipate most of their heat into the outer space, although they cool slowly, but can achieve a much higher temperature reduction once steady state is reached.

In addition, as the atmospheric transparency of the second atmospheric window is slightly less than 1 compared with mono-selective emitters, there is also an additional (albeit small) atmospheric heating effect for dual-selective emitters that cannot be ignored under ideal conditions. Therefore, in ideal environments, mono-selective thermal emitters have a slightly higher temperature reduction than dual-selective emitters.

Supplementary text 3. Effect of non-radiative heat on the subambient cooling performance of dual-selective, mono-selective, and mono-selective thermal emitters.

In real scenarios ($h \geq 1$ W m^{-2} K^{-1}), the presence of non-radiative heat (*e.g.*, convection) will induce heat exchange between the radiative cooling system and the surroundings, counteracting the radiative cooling effect (*i.e.*, heat leakage) and drastically weakening the subambient cooling performance of thermal emitters (Fig. 1c,e and Supplementary Fig. 2). Importantly, the higher the net cooling power (the faster they dissipate heat), the less the subambient cooling performance of a thermal emitter is affected by non-radiative heat effects.

Comparing mono-selective thermal emitters with non-selective thermal emitters, when the non-radiative effect is small (h = 1–2 W m^{-2} K^{-1}), the subambient cooling capacity of the former is greater than that of the latter due to the fact that the former can exclude atmospheric parasitic heat and is subject to less attenuation by non-radiative heat. If the non-radiative effect is significant ($h \geq 3$ W m^{-2} K^{-1}), the attenuation of the subambient cooling capacity of the former is much greater than that of the latter due to its much lower net cooling power than that of the latter, resulting in the subambient cooling performance of the former being less than that of the latter.

With regard to dual- and mono-selective thermal emitters, although both are capable of eliminating atmospheric parasitic heat, due to the much higher net cooling power of the former, the attenuation of the subambient cooling capacity of the former affected by non-radiative heat is much less than that of the latter. Therefore, in real environments ($h \geq 1$ W m^{-2} K^{-1}), dual-selective thermal emitters have a much better subambient cooling performance than mono-selective thermal emitters.

In addition, when comparing the dual-selective with non-selective thermal emitters, although they have relatively similar net cooling power (*i.e.*, relatively similar heat dissipation rate), the

former can exclude atmospheric parasitic heat. Therefore, dual-selective thermal emitters have a better subambient cooling performance than non-selective thermal emitters in real environments (h = 1–6 W m^{-2} K^{-1}).

In summary, dual-selective thermal emitters can exclude atmospheric parasitic heat and exhibit high cooling power, combining the advantages of non-selective and mono-selective thermal emitters and avoiding their disadvantages (inability to exclude atmospheric parasitic heat for non-selective thermal emitters and low net cooling power for mono-selective thermal emitters). As a result, in real arid environments, the subambient radiative cooling performance of dual-selective thermal emitters is notably better than that of non-selective and mono-selective counterparts.

Supplementary text 4. Modelling of the effect of humidity on the radiative cooling performance of non-selective, mono-selective, and dual-selective RC models

To evaluate the effect of humidity on the radiative cooling performance of dual-, mono-, and non-selective RC models, in addition to the arid (low humidity) environment, we also simulated their cooling performance in a higher humidity environment (with different non-radiative heat effects, h = 0, 1, 2, 4, 6 W m^{-2} K^{-1}, Supplementary Figs. 6,7). The atmospheric transparency data in a high humidity environment for the simulation were the same with the "Sub-humid" data in Fig. 1b, as shown in Supplementary Fig. 7a. As can be seen, the atmospheric transparency in the second atmospheric window waveband (16–25 μm) is much reduced compared with the low humidity data due to the strong heat absorption by water in this waveband.

The theoretical calculations show that in a high humidity environment, the mono-selective RC model shows optimal subambient cooling performance in both ideal (h = 0 W m^{-2} K^{-1}) and real ($h \geq 1$ W m^{-2} K^{-1}) scenarios (Supplementary Figs. 6a–e,7b), which is very different from the cooling performance in low humidity conditions (Fig. 1e). More importantly, in real scenarios, the dual-selective model shows a subambient cooling performance close to the mono-selective model (especially for h = 2–6 W m^{-2} K^{-1}) and better than the non-selective model (1.3–7.8 °C cooler) (Supplementary Fig. 7b), which results from its higher cooling power (71.8–136.5 W m^{-2}, $T_{ambient}$ = 0–40 °C) than the mono-selective model (67.8–130.6 W m^{-2}) (Supplementary Fig. 6f) and less atmospheric parasitic heat than the non-selective model.

Furthermore, compared with the low humidity environment, the net cooling power and subambient temperature reduction are notably reduced in the high humidity environment (Supplementary Figs. 7c–f) due to the fact that the atmospheric transparency of the atmosphere is notably reduced as the ambient humidity increases, resulting in a low heat dissipation efficiency.

It is worth noting that the cooling performance decrease of the mono-selective RC model with increasing humidity is significantly smaller than that of the other two RC models. Specifically, the cooling power reduction of the mono-selective model is only 12.9–25.1 W m^{-2} ($T_{ambient}$, 0–40 °C), whereas the reductions for the dual-selective and non-selective models reach 49.1–77.7 W m^{-2} and 59.6–102.9 W m^{-2}, respectively (Supplementary Fig. 7c). Moreover, in real scenarios (h = 1–6 W m^{-2} K^{-1}), the decrease of the subambient cooling temperature of the mono-selective model is only 2–8 °C, whereas the cooling temperature reduction of the other two RC models reaches 7–20 °C (Supplementary Figs. 7d–f). This is due to the fact that with the humidity increase, the reduction in atmospheric transparency in the main atmospheric window (8–13 μm) is much less than the reduction in the second window (16–25 μm) (Supplementary Fig. 7a). Therefore, the degradation in cooling performance of the mono-selective model is much less than that of the dual- and non-selective models.

These results show that with the ambient humidity increase, the cooling performance of all three RC models decreases accordingly, but the dual-selective model still maintains a subambient cooling performance close to the mono-selective RC model and notably better than the non-selective RC model. Importantly, with the ambient humidity decrease, the cooling performance increase of the dual-selective RC model is significantly larger than that of the mono-selective RC model, indicating that the former has a better subambient cooling performance than the latter in real scenarios.

Supplementary text 5 Optical modeling of POM fibers and PTFE particles

The optical simulations were based on the Mie theory together with Chandrasekhar radiative transfer theory[4–6]. The scattering efficiency of POM fibers and PTFE particles was simulated over the wavelength range of 0.3–2.5 μm with the POM fiber diameter varying from 0.2 to 2 μm and the PTFE particle diameter varying from 0.2 to 3 μm (Fig. 2c).

In previous work on radiative cooling, the calculations of the scattering properties in the 0.3–2.5 μm waveband often ignored the absorption of the substrate and defined the refractive index as a constant[7,8]. The corresponding refractive indexes of POM and PTFE were extracted from previous works[9–11].

Supplementary text 6. Design principles for the diameter distribution of PTFE particles and POM fibers

According to the Mie scattering theory, the diameter distribution of the particles or fibers is the key to achieving a high solar reflectance of a particle- or fiber- based thermal emitter (for daytime radiative cooling). We have selected the appropriate size distribution of PTFE particles and POM fibers according to the following principles.

1) First, according to our theoretical calculations for PTFE particles based on the Mie scattering theory (Fig. 2b), for a given thickness of a radiative cooler, the highest solar reflectance can be achieved in an air medium when the size distribution of PTFE particles is around 0.2–3.0 μm (which is close to the solar waveband). It should be noted that the result was obtained for PTFE particles in an air medium where there is a large difference in refractive indices between PTFE and air, resulting in strong scattering at the polymer/air interface. However, for the POM-PTFE complexes-based samples, considering the matching of POM fibers and PTFE particles in the samples, we prefer to select PTFE particles with a size larger than that of POM fibers. The reason is that the as-used polymers, *i.e.*, PTFE and POM, both have similar refractive indices (~1.5)[9,11,12]. If the size of the PTFE particle is similar or smaller than that of the POM fiber (*e.g.*, using nano-sized PTFE particles to prepare the POM-PTFE electrospun film), the Mie scattering effect of the particles will be masked by the POM fibers because the PTFE nanoparticles are embedded in the POM fibers (*i.e.*, the PTFE particles are not in the air medium). Therefore, the PTFE particles with a larger size distribution than that of the POM fibers were used to prepare the dual-selective samples.

2) In adiation, our previous work has shown that nano-sized POM fibers with diameter distribution close to the main waveband of sunlight (0.2–1.0 μm) can be easily produced by electrospinning[13].

3) Finally, nanoparticle-based products are susceptible to health risks due to the inevitable inhalation of the human body during production and use. Besides, although both nano- and micron-sized PTFE particles are commercially available, nano-sized PTFE particles are much more expensive than micron-sized PTFE particles.

Therefore, taking the Mie scattering effect, compatibility with POM fibers, safety, and cost of PTFE particles into account, the POM fibers with distribution of 0.2–1.0 μm and PTFE particles with distribution of 1.0–3.0 μm were used to prepare the dual-selective samples.

As an additional note, when changing the size of the PTFE particles (in this work it refers to reducing the size of the PTFE particles from the micro size distribution to the nano size distribution), it is necessary to prepare the POM fibers with larger diameters according to the Mie scattering theory (centered around 600 nm, Fig. 2b), so that their diameter distribution is closer to the main wavebands of sunlight. The larger diameter of electrospun POM fibers can be achieved in one or more of the following ways: increasing the concentration of the electrospinning solution, decreasing the spinning voltage, and decreasing the distance between the positive and negative electrodes[14–18].

Supplementary text 7. Effect of thickness on optical properties of the POM-PTFE-Al

Due to the inevitable broadening of the molecular vibrations, an increase in thickness will result in a decrease in the spectral selectivity of the thermal emitter in the two atmospheric window wavebands, whereas a sufficiently thick thickness is required for a high solar reflectance. Therefore, to obtain a dual selective thermal emitter with high spectral selectivity and high solar reflectance, an appropriate thickness to achieve the trade-off between high selectivity and high solar reflectance is critical. Based on the suitable functional groups (C-O-C and C-F) and micro-nano structures (POM nanofiber-PTFE microparticle bead-like fibers), the spectral response of the POM-PTFE-Al was also studied and optimized by tuning its thickness to fit the dual-selective RC model, which requires both high solar reflectance, high selective emittance in the two atmospheric window wavebands (8–13 μm and 16–25 μm, respectively), and low emittance in non-window MIR wavebands.

Based on the above analysis, a principle of thickness selection is to keep the thickness as thin as possible while ensuring a high solar reflectance (≥95%). As shown in Supplementary Fig. 14, the solar reflectance of the POM-PTFE-Al gradually increased with increasing its thickness (92.0%, 95.4% and 95.9% for the thicknesses of 100, 150, and 200 μm, respectively). To balance the requirements between high solar reflectance (≥95%) and high selective emission characteristics, the thickness was finally determined to be ~150 μm.

Supplementary text 8. Effect of the mass ratio of PTFE/POM on optical properties of the POM-PTFE-Al

Similar to the thickness, the content of PTFE micron particles and POM nanofibers in the POM-PTFE two-component polymer is also crucial to achieve the desired dual-selective properties. Based on the suitable thickness (150 μm) & micro-nano structures (bead-like fibers) (a high solar reflectance can be achieved), and the carefully selected functional groups (C-O-C and C-F), the MIR spectral response of the POM-PTFE-Al was also studied and optimized by tuning the PTFE content to match the dual selective properties. As shown in Supplementary Fig. 15, the emittance of the POM-PTFE-Al in the two atmospheric windows gradually increased with increasing the PTFE content (20%~60%), while the spectral selectivity of the thermal emitter in the two windows was sufficiently reduced. To balance the requirements of the high window emittance and high selective emission characteristics, the PTFE content of POM-PTFE was finally determined to be 30%. As a result, a 150 μm-thick thermal emitter with 30wt% PTFE exhibited a high solar reflectance up to 95.4% in the 0.3–2.5 μm waveband and a high selective MIR emissivity in both atmospheric windows (83.2% in the first atmospheric window (8–13 μm) and 67.5% in the second window (16–25 μm), respectively), which is much higher than that in the non-windowed waveband (~45%), indicating a desired dual-selective characteristic.

Supplementary text 9. Calculation of estimated UV aging time of the dual-selective sample in Beijing

The estimated UV aging time was calculated according the previous reported methods[19]. Specifically, the annual solar irradiation time and the total solar irradiation power in Beijing are ~2600 h and 1509 kWh m^{-2} $year^{-1}$), respectively. The total annual UV radiation in this work was calculated to be 75450 Wh m^{-2} $year^{-1}$ (5% of total solar irradiation). The radiation power under test conditions was 125 W m^{-2}. Thus, estimated that the simulated irradiation was about 4.31 times higher than that of outdoor UV irradiation (125 W m^{-2} ÷ 75450 Wh m^{-2} $year^{-1}$ ÷ 2600 h $year^{-1}$ ≈ 4.31). In addition, the annual average temperature in Beijing is 11.6 °C. The empirical aging effect evaluation will increase by about 1.5 times when the temperature increases by 1 time. The actual test temperature was greater than 60 °C, we set it to be 60 °C. Based on the above analysis, we obtained a relationship between the estimated UV aging time and the accelerated UV-aging time 4.31*60/11.6*1.5 = 33.42. As the actual time of UV irradiation test in this work was 300~500 h, and the annual outdoor sunlight irradiation time is about 8 hours per day (considering the removal of cloudy or rainy days), therefore, the total estimated UV aging time is about (300~500 h) *33.42 *24/8 = 30078~50130 h (which equals to 1253~2089 days, more than 3~5 years).

Supplementary text 10. Selection of the mono-selective and non-selective thermal emitters

To demonstrate the enhanced cooling performance of dual selective emitters over existing typical designs, a comparison of our dual selective sample with typical mono-selective and non-selective thermal emitters will serve the purpose.

Due to the pure C-O-C bond of POM, POM nanofiber film covered on Al foil (donated as POM-Al) is a desirable candidate for mono-selective thermal emitters, as the strong vibrations of the C-O-C bond are mainly located in the first atmospheric window (8–13 μm) and the weak vibrations in the remaining MIR waveband (Fig.2a and Supplementary Fig. 12). After further careful thickness control, a 120 μm-thick POM-Al with similar solar reflectance (94.3%) to the dual-selective POM-PTFE-Al and the desired mono-selective characteristics (77.3% in the 8–13 μm waveband, ~32% in the remaining MIR wavebands) was obtained, and it was used for comparison with the dual-selective sample.

Previous work has shown that PVDF is a potential non-selective thermal emitter due to its strong absorption/vibration peaks throughout the MIR waveband (Supplementary Fig. 26)[20–22]. After careful regulation in our previous work[13], a ~300 μm-thick PVDF electrospun film combined both high solar reflectance (95.1%, similar to dual-selective POM-PTFE-Al) and significant non-selective characteristics (high emissivity of ~90% in the 4–25 μm band), further demonstrating that it is an ideal non-selective emitter.

Supplementary text 11. Experimental comparison of the net cooling power between the non-selective sample and the dual-selective sample.

As shown in the simulations in this work (Fig.1), dual-selective thermal emitters exhibit higher subambient cooling performance than mono- and non-selective counterparts in real arid environments. However, the reasons why their subambient cooling performance is higher than that of the other two types of thermal emitters are different. Specifically, the higher performance than mono-selective thermal emitters is due to that dual-selective emitters have a much higher net cooling power than mono-selective thermal emitters, which has been demonstrated by the cooling power measurements (Fig. 4b). In contrast, the higher performance of dual-selective thermal emitters compared with non-selective thermal emitters is because of the ability of dual-selective emitters to exclude atmospheric parasitic heat, not because of their higher net cooling power (which is in fact slightly lower than that of non-selective thermal emitters). This is also the reason why the cooling power data of the non-selective sample is not included in Fig. 4b. To demonstrate this, we also compared experimentally the net cooling power of the non-selective and dual-selective samples in a similar arid environment (RH = ~12%, Supplementary Figs. 34 and 35). The results show that under strong solar irradiation (~700 W m^{-2}), the dual-selective POM-PTFE-Al exhibited slightly lower net cooling power (131.2±19.7 W m^{-2}), than the non-selective PVDF (154.5±11.0 W m^{-2}), in agreement with our simulations.

It is worth noting that the cooling power of the dual-selective sample here is lower than the result in Fig. 4b (151.8 ± 13.1 W m^{-2}, Fig. 4b), which is due to the two tests with different environmental conditions between the two cooling power measurements, including ambient temperature, humidity, wind speed, and solar irradiation (mainly lower temperatures and higher wind speeds, resulting in lower net cooling power).

Supplementary text 12. Correlation between the simulated and the measured cooling powers

To explore the potential correlations between the simulated and measured results for the net cooling powers, we have simulated the net cooling powers of these samples according to the ambient temperature of the real measurement conditions and compared them with the real experimental results.

As shown in Supplementary Fig. 33b, the measurements for the dual-selective and mono-selective samples ($T_{ambient}$ = ~38 °C, 7 September 2022) showed that the dual-selective POM-PTFE-Al exhibited an ultra-high net cooling power (151.8±13.1 W m^{-2}), which was much larger than that of the mono-selective POM (87.9±9.4 W m^{-2}). Their corresponding theoretical cooling powers (excluding solar heat and $T_{ambient}$ = 38 °C) were 208.8 W m^{-2} and 151.2 W m^{-2}, respectively. Taking the cooling power compensated by solar heat into consideration (840 W m^{-2} for solar irradiation, 38.6 W m^{-2} for the dual-selective sample and 47.9 W m^{-2} for the mono-selective sample), the actual measured net cooling powers of the dual-selective and mono-selective samples were 190.4±13.1 W m^{-2} and 135.8±9.4 W m^{-2}, respectively, which were close to the theoretical values.

As shown in Supplementary Fig. 35b, the measurements for the dual-selective POM-PTFE-Al and non-selective PVDF in an environment with lower ambient temperature ($T_{ambient}$ = ~31 °C, 9 September 2022) showed that the dual-selective sample exhibited a high net cooling power (131.2±19.7 W m^{-2}), which was close to that of the non-selective samples (154.5±11.0 W m^{-2}). Their corresponding theoretical cooling powers (excluding solar heat and $T_{ambient}$ = 31 °C) were 190.4 W m^{-2} and 221.0 W m^{-2}, respectively. Taking into account the cooling power compensated by solar heat (700 W m^{-2} for solar irradiation, 32.2 W m^{-2} for the dual-selective sample and 34.3 W m^{-2} for the non-selective sample), the actual measured net cooling powers of the dual-selective

and mono-selective samples were 163.4±19.7 W m^{-2} and 188.8±11.0 W m^{-2}, respectively, which were also close to their theoretical values.

In conclusion, our experimental results are lower than the theoretical values, mainly due to the absorption of solar heat. When the solar heating effect is taken into account, they are close to the theoretical values. Nevertheless, there are still deviations between the theoretical and experimental values due to unavoidable deviations, such as humidity, wind and instrumentation conditions in the actual tests.

Supplementary text 13. Measurement of the radiative cooling performance of the blue dual-selective thermal emitter.

To demonstrate the radiative cooling performance of the colored dual-selective thermal emitters, in addition to the yellow dual-selective sample, the cooling performance of the blue dual-selective sample was tested and compared with the commercial counterpart (blue PE covered commercial white paint) in Beijing, China (8 October 2023). The IR camera tests in a low-humidity environment (RH ~18% and Solar ~500 W m^{-2}, Supplementary Figs. 52 and 53) showed that, under strong sunlight, the blue PE covered dual-selective POM-PTFE-Al thermal emitter exhibited significantly lower cooling performance (5–8 °C cooler) than the blue PE covered commercial white paint (Supplementary Figs. 53b and Supplementary movie 5). These results showed that although subambient cooling could not be achieved, the blue sample still had significantly better cooling performance than the commercial counterpart due to the above-ambient radiative cooling effect.

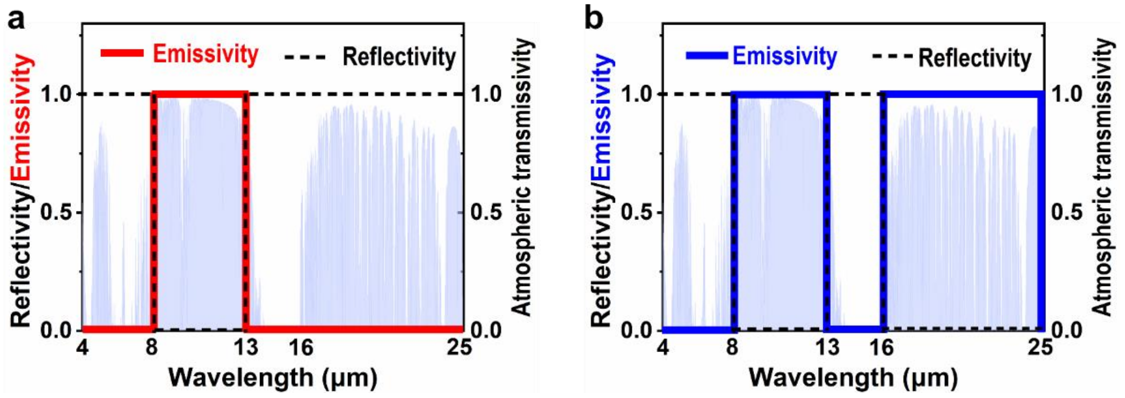

Supplementary Fig. 1. Spectral features in the MIR region for the mono-selective (a) and dual-selective (b) RC model.

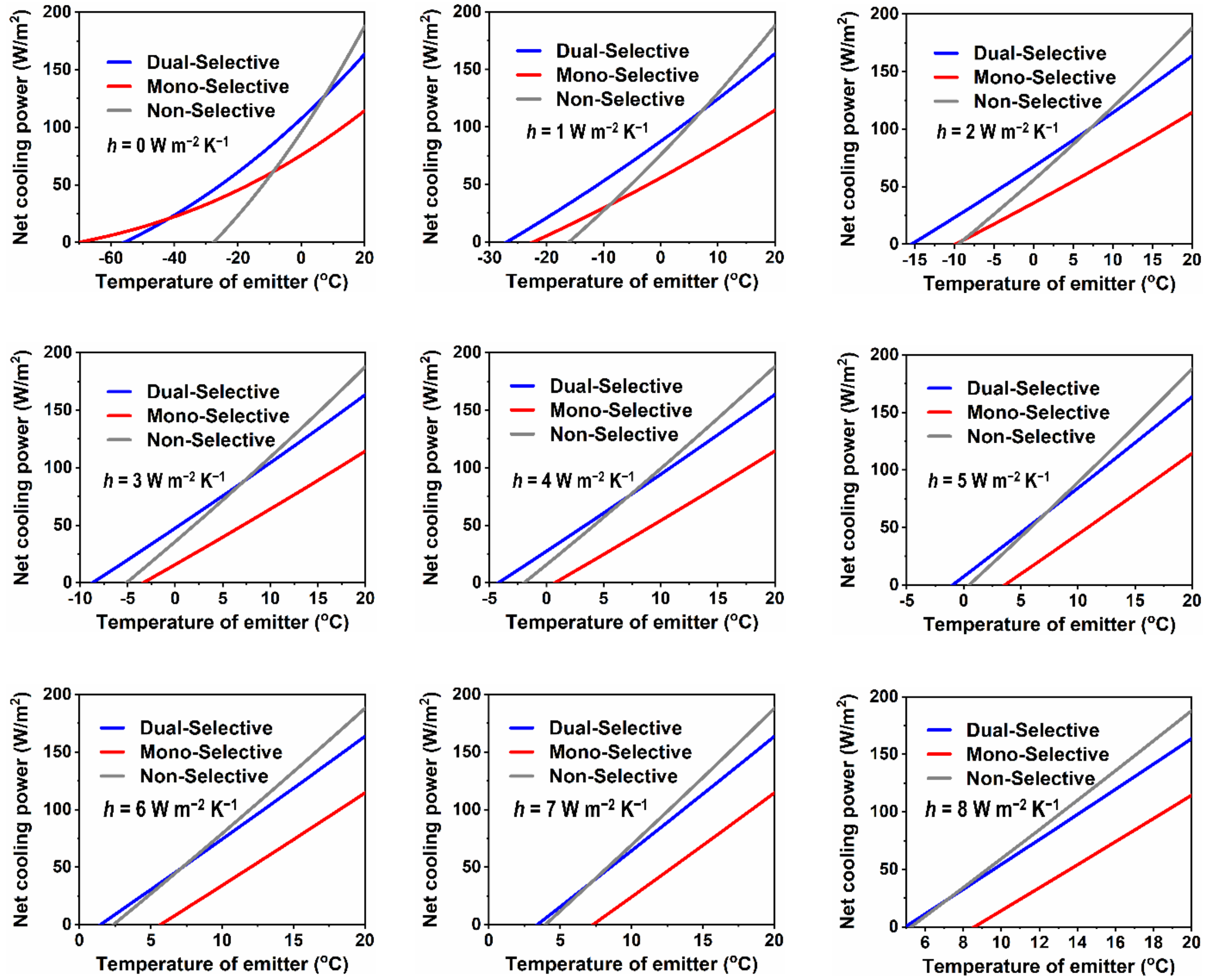

Supplementary Fig. 2. Theoretical cooling power of the three different radiative coolers as a function of the emitter temperature in an arid environment (T_{ambient} = 20 °C). The theoretical limit of subambient cooling (steady-state temperature) of the sample is reached when the cooling power reaches zero, corresponding to the intersection of the curve with the *x*-axis in these figures, which is the source of the data in Fig. 1e and Supplementary Fig. 3. The instinctive cooling power of the sample (as shown in Fig. 1d) is reached when there is no temperature difference between the sample and the ambient, corresponding to the intersection of the curve with the *y*-axis in these figures.

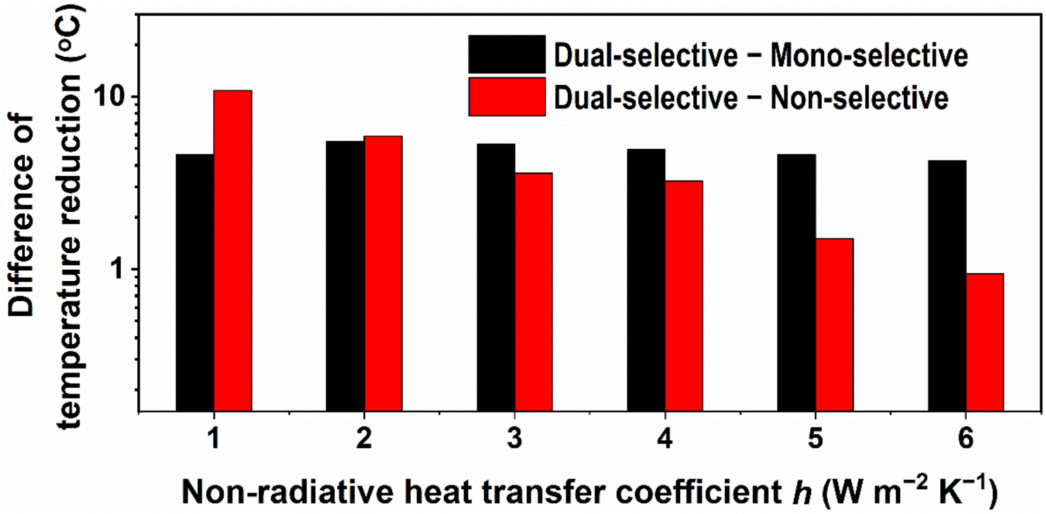

Supplementary Fig. 3. Difference in the theoretical temperature reduction compared to the dual-selective radiative cooler at different non-radiative heat transfer coefficients h (1–6 W m^{-2} K^{-1}) for the mono-selective and non-selective radiative coolers at an ambient temperature of 20 °C.

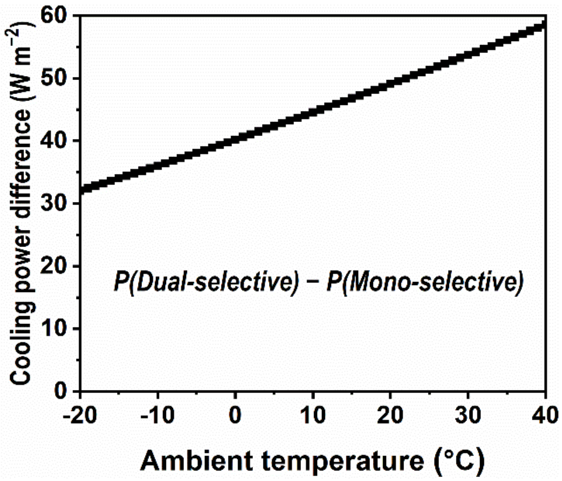

Supplementary Fig. 4. Difference of the theoretical cooling power between the dual-selective and mono-selective radiative coolers at different ambient temperatures (−20° to 40 °C). The calculation is based on the condition that the temperature of the thermal emitter is equal to the ambient temperature.

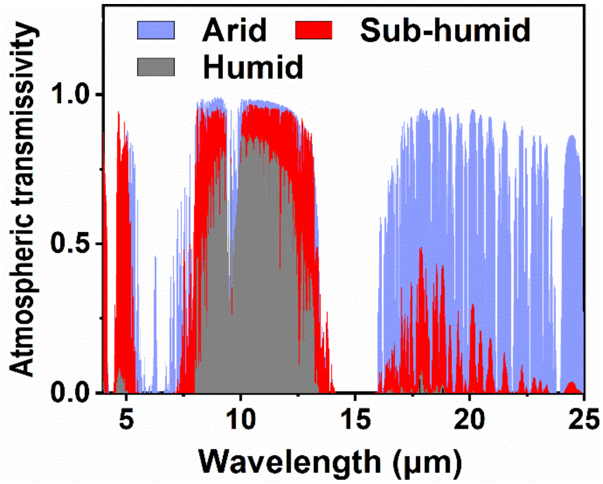

Supplementary Fig. 5. Atmospheric transmissivity of arid (low RH), sub-humid (normal RH), and humid (high RH) climates.

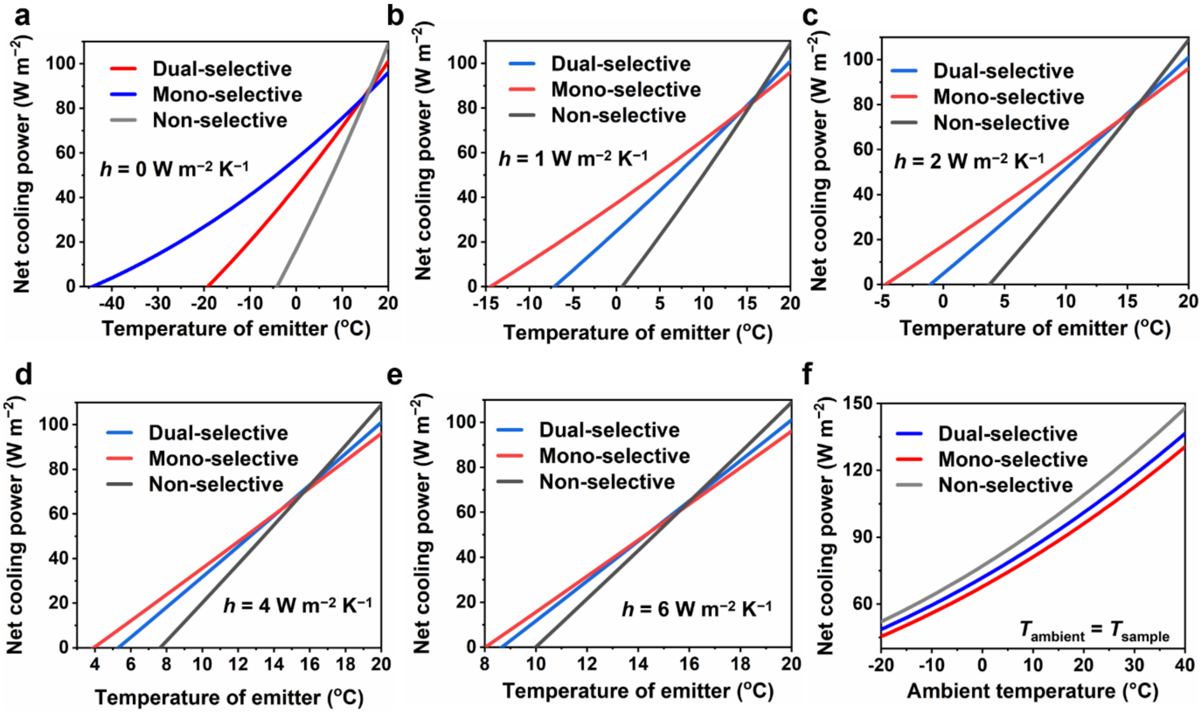

Supplementary Fig. 6. Theoretical cooling performance of the three different radiative coolers in a high humidity environment. **a–e,** Cooling power of the three different radiative coolers as a function of the emitter temperature in an arid environment ($T_{ambient}$ = 20 °C). **f**, Comparison of the theoretical cooling power of the three different radiative coolers at different $T_{ambient}$ (−20° to 40 °C). The calculation is based on the same atmospheric transmittance data with the "sub-humid" in Supplementary Fig. 5. Source data are provided as a Source Data file.

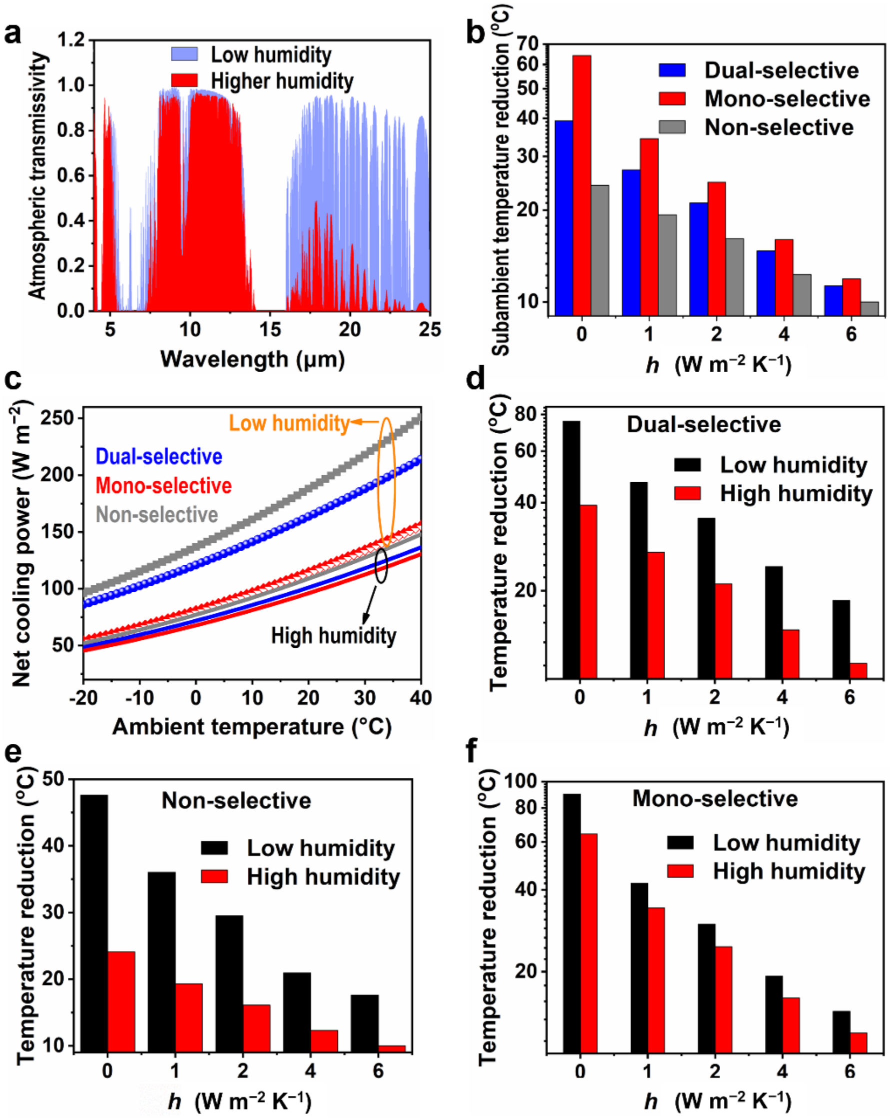

Supplementary Fig. 7. Comparison of cooling performance of the three different radiative coolers between low humidity and higher humidity. a, Atmospheric transmittance data of low and high humidity environments. **b**, Theoretical limit of subambient cooling of the three types of radiative coolers at different h (0–6 W m^{-2} K^{-1}). The calculation is based on the atmospheric transmittance data in high humidity, a net cooling power P_{net} of 0 W m^{-2} and T_{ambient} = 20 °C. **c–f**, Comparison of cooling power (c) and subambient temperature reduction of the three different radiative coolers (d–f) between low humidity and higher humidity.

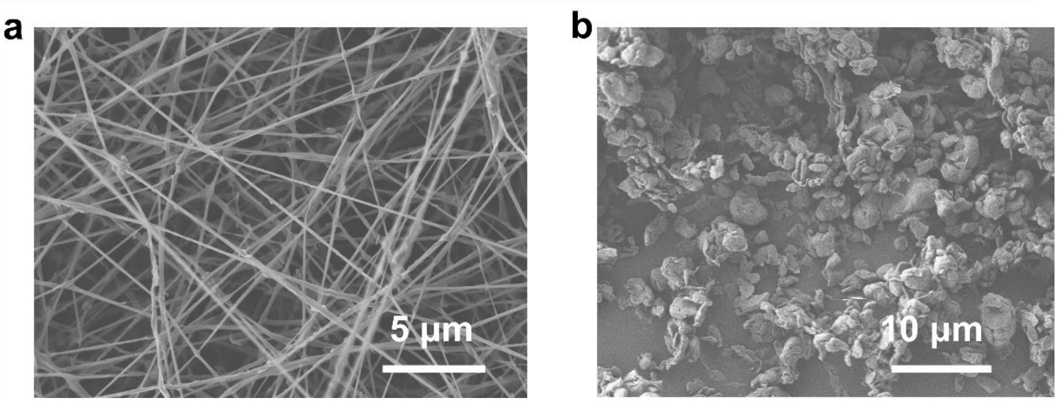

Supplementary Fig. 8. SEM images. a, POM nanofibers prepared through an electrospinning process (details see **Methods** section). **b,** Commercial PTFE micro-particle.

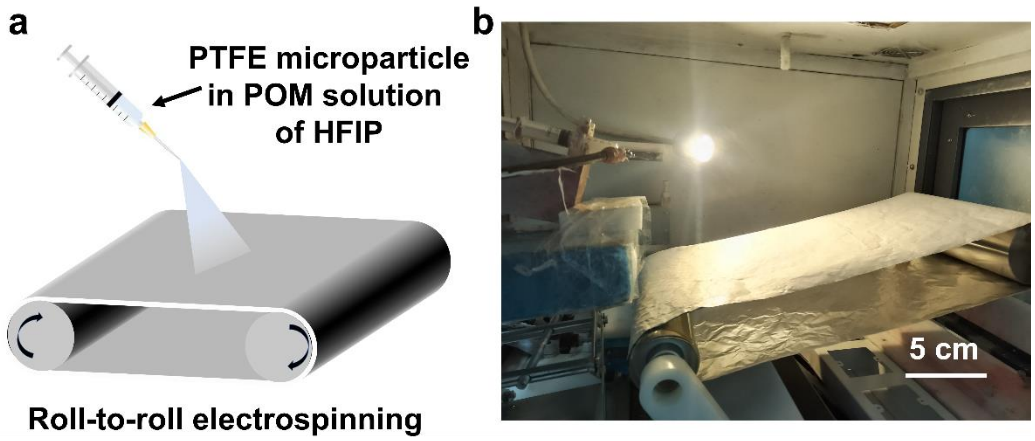

Supplementary Fig. 9. Fabrication of the POM-PTFE film through a roll-to-roll electrospinning. a and **b**, Schematic (**a**) and digital image (**b**) of the electrospinning process.

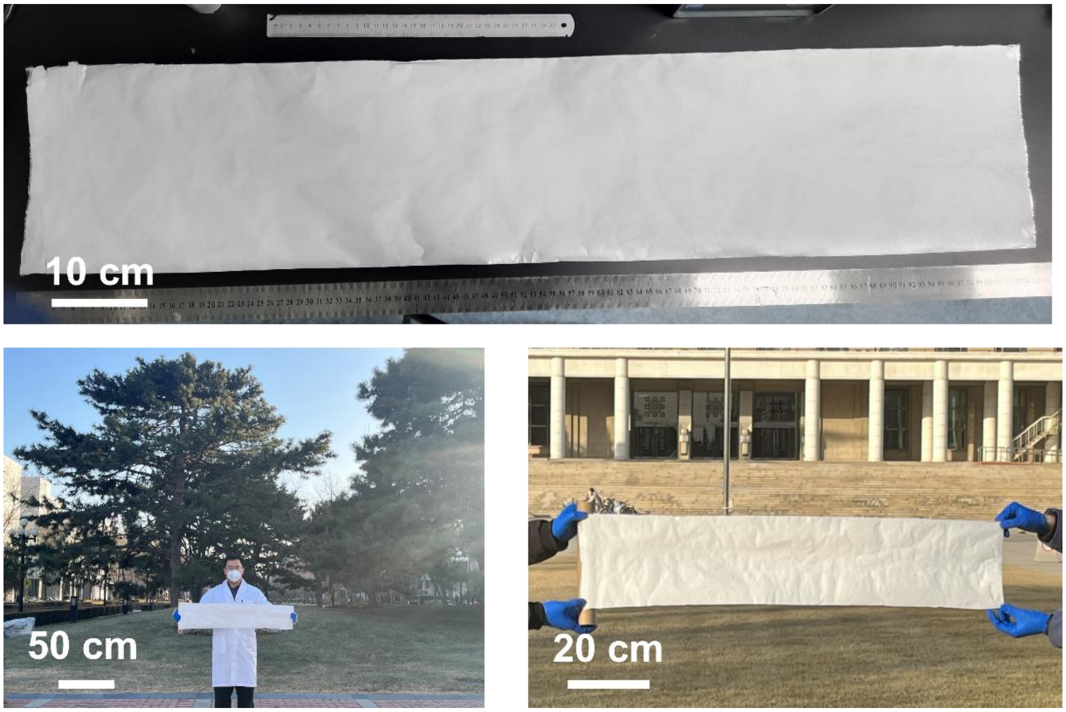

Supplementary Fig. 10. Digital images of the POM-PTFE films prepared through an electrospinning process.

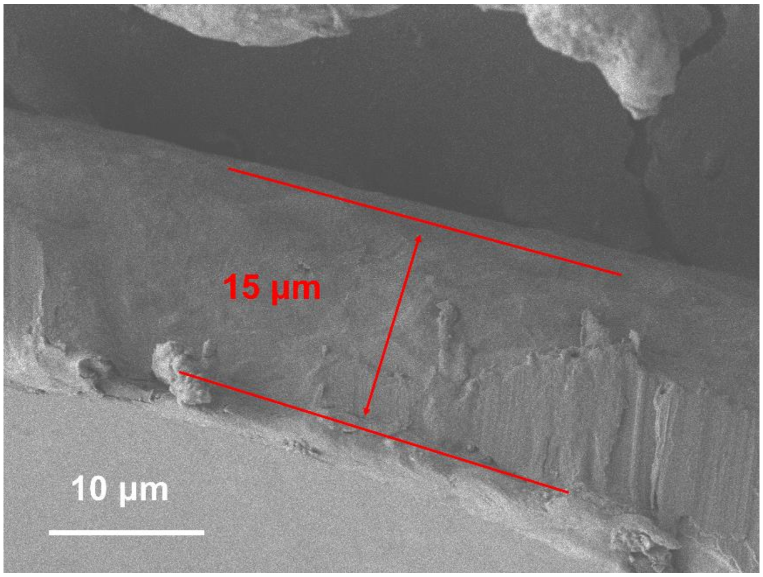

Supplementary Fig. 11. Cross-sectional SEM image of an Al film (with a thickness of~15 μm).

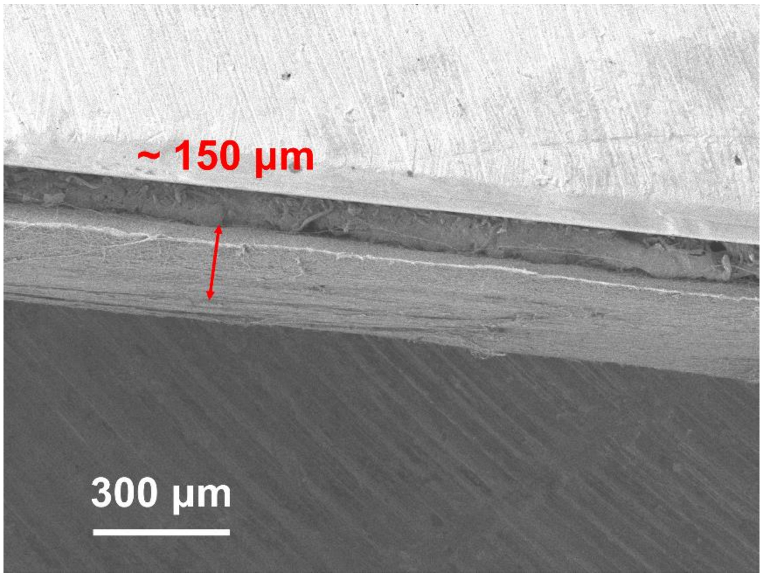

Supplementary Fig. 12. Cross-sectional SEM image of a dual-selective POM-PTFE film. The thickness of the POM-PTFE film is ~150 μm.

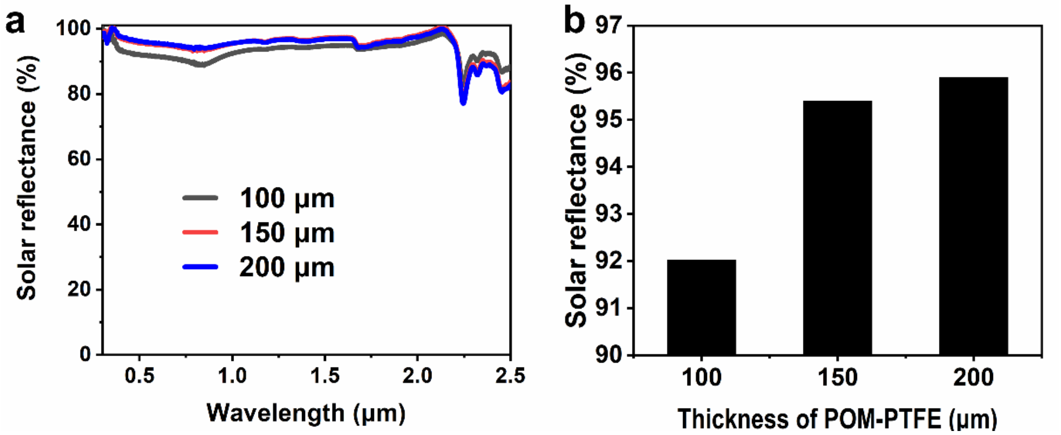

Supplementary Fig. 13. Spectral response of the POM-PTFE-Al film with different POM-PTFE film thicknesses. Solar reflectance spectra (**a**) and the corresponding average solar reflectance (**b**). Source data are provided as a Source Data file.

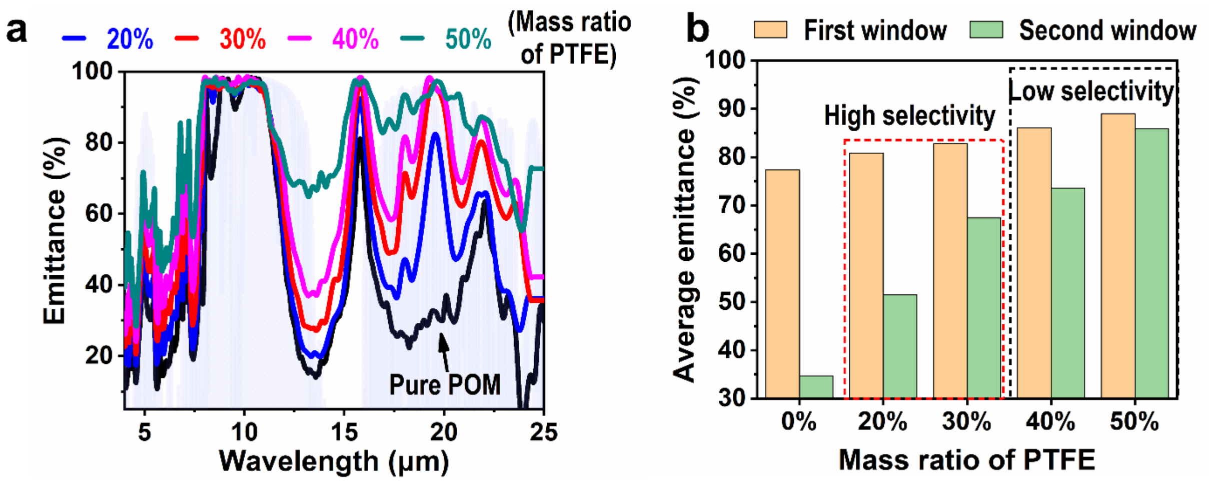

Supplementary Fig. 14. Spectral response of POM-PTFE-Al film with different PTFE content (mass concentration). a, MIR emittance spectra. **b**, Corresponding average MIR emittance in the first (8–13 μm) and second (16–25 μm) atmospheric windows. Source data are provided as a Source Data file.

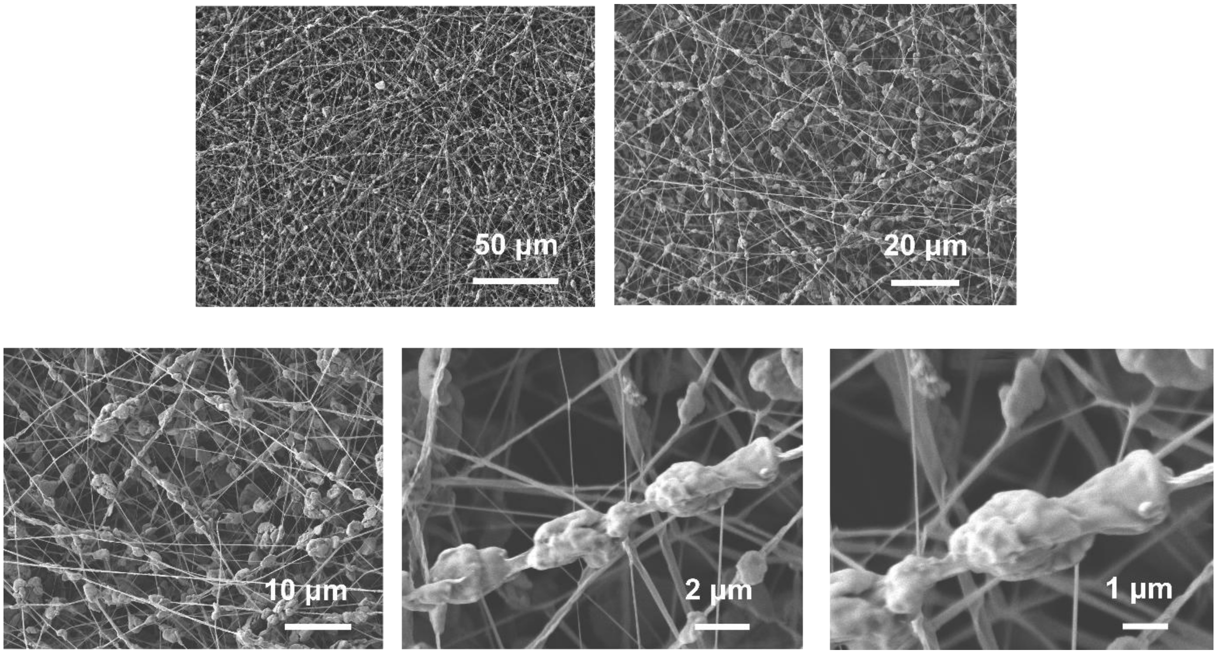

Supplementary Fig. 15. SEM images of a dual-selective POM-PTFE film, which has a bead-like fiber structure consisting of POM nanofibers and PTFE micron particles.

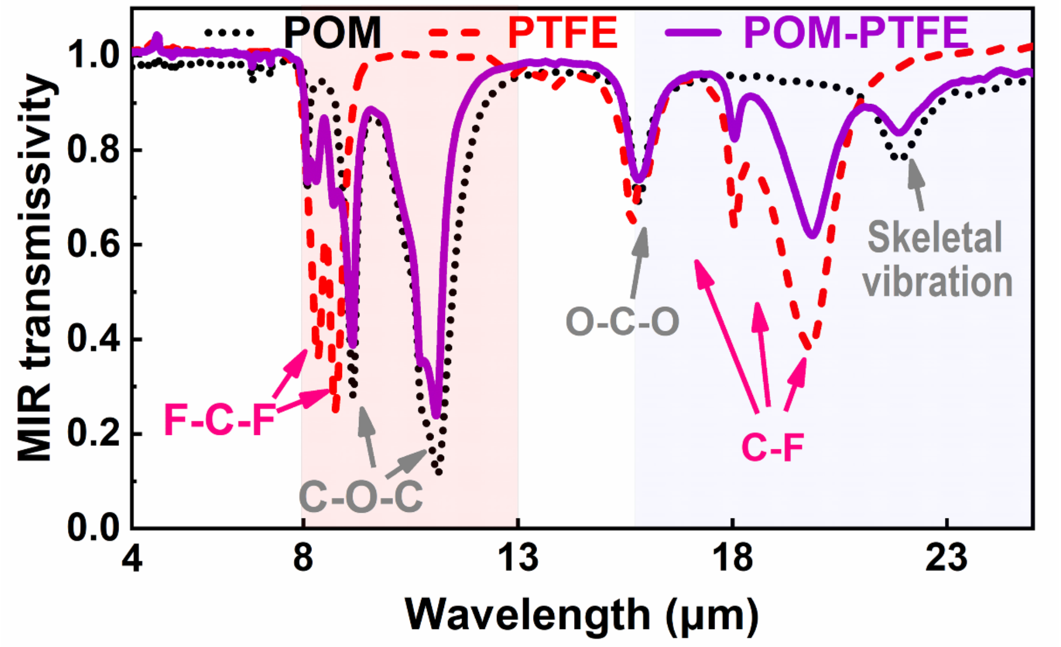

Supplementary Fig. 16. FTIR-ATR spectra of POM, PTFE and dual-selective POM-PTFE where the main characteristic peaks of C-O-C vibrational absorption/emission of POM are located in the region of the first atmospheric window (8–13 μm), and the C-F vibrational absorption/emission of PTFE mainly in the second atmospheric window (16–25 μm). The figure is intended to compare the experimentally prepared POM-PTFE sample with the previous molecular design, to show that the experimental results and the molecular design are in agreement.

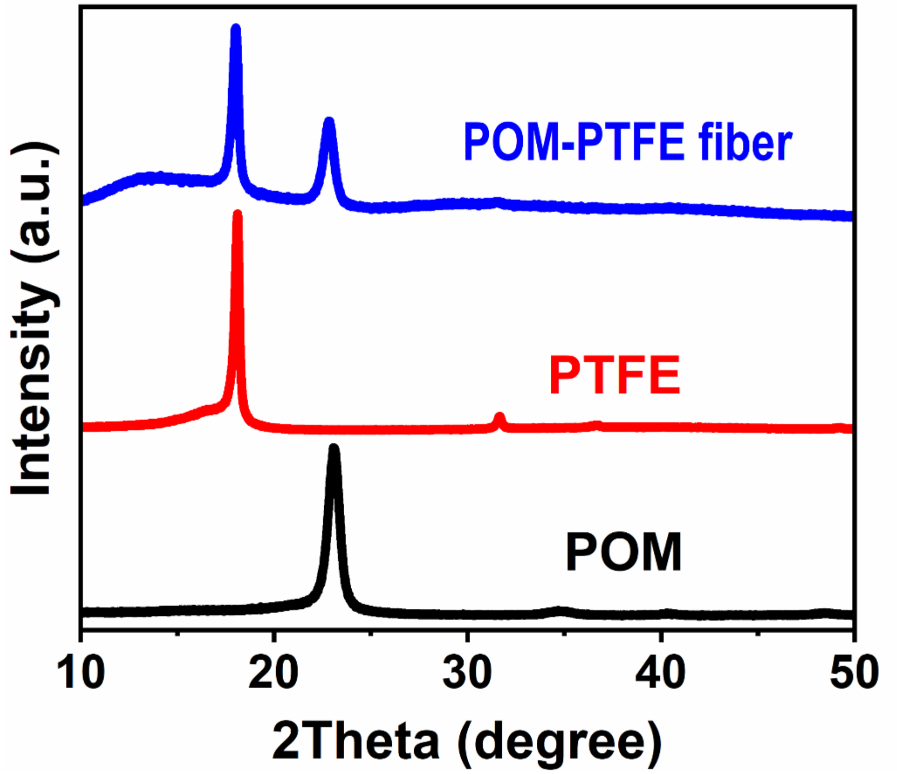

Supplementary Fig. 17. XRD patterns of an electrospun POM film, PTFE powder, and a dual-selective electrospun POM-PTFE film.

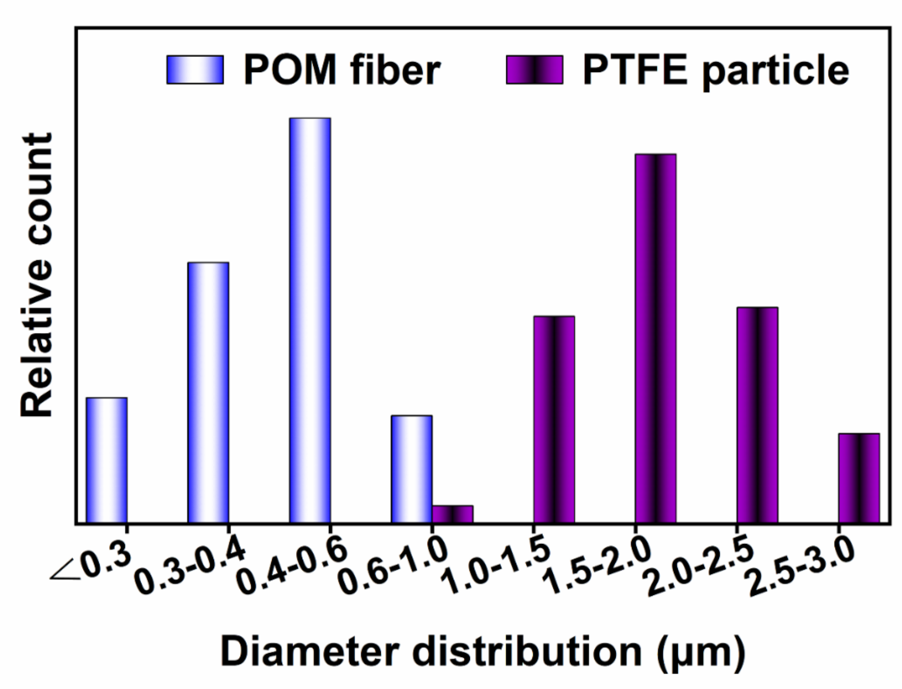

Supplementary Fig. 18. Statistical distribution of the diameters of the POM nanofibers and PTFE particles inside the dual-selective POM-PTFE film. ImageJ software was used to perform these statistics based on the SEM images of the POM-PTFE electrospun film (Supplementary Fig. 15). The number of samples of both fibers and particles was 100 (randomly selected from the SEM images), and the ratio of the number of particles/fibers to the total number of samples in each size range was counted *(i.e.*, relative count), so that the size distribution of fibers and particles could be obtained directly. The final average diameter values obtained for POM fibers and PTFE particles are the average sizes of the 100 samples measured with ImageJ, which are 502 ± 154 nm and 1.8 ± 0.49 μm, respectively.

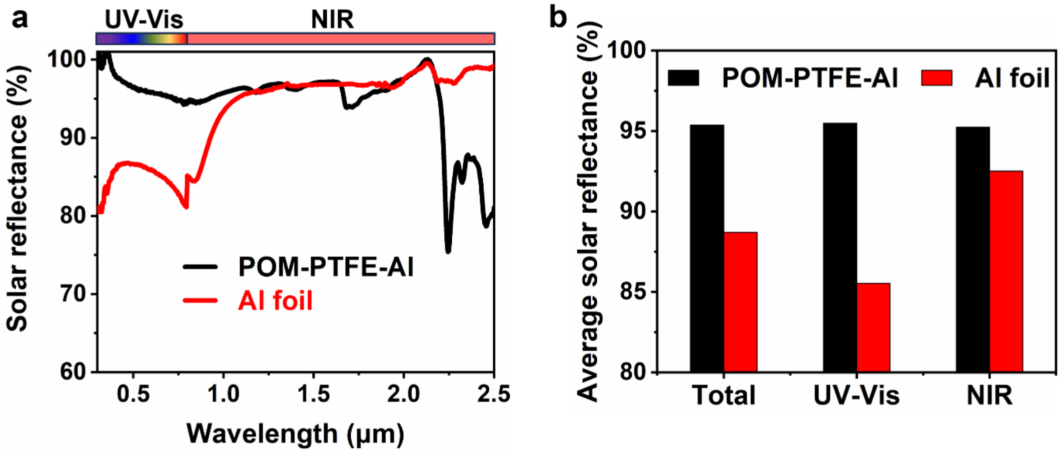

Supplementary Fig. 19. Comparison of the solar reflectance spectra of the POM-PTFE-Al sample and the pure Al foil in the 0.3–2.5 μm waveband. a, Solar reflectance spectra of both the POM-PTFE-Al and the Al foil. **b**, Corresponding average reflectance of total (0.3–2.5 μm), UV-vis (0.3–0.76 μm), and NIR (0.76–2.5 μm) wavebands, respectively. As can be seen, the POM-PTFE-Al sample showed a much higher solar reflectance (95.4%) than that of the pure Al foil (88.7%), indicating that the POM-PTFE electrospun film was indeed effective in enhancing the solar reflectance of the dual-selective sample. The further analyzes show that its higher solar reflectance was due to its higher reflectance in both the ultraviolet-visible (UV-Vis, 95.5%) and the near-infrared (NIR, 95.2%) wavebands than the pure Al foil (85.5% and 92.5%, respectively). The much higher UV-Vis reflectance (0.3–0.76 μm) of POM-PTFE-Al than the pure Al foil was mainly due to the nano-design of the POM nanofibers and the higher NIR reflectance (0.76–2.5 μm) was mainly due to the micro-design of the PTFE microparticles. These results demonstrate that the enhancement of the solar reflectance by the nano/micron structure (including POM nanofibers and PTFE microparticles) determined from Mie scattering simulations is remarkable.

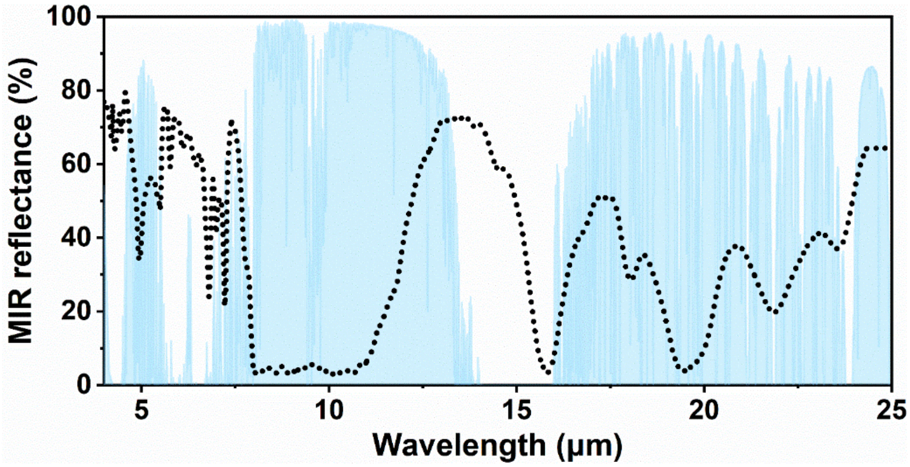

Supplementary Fig. 20. MIR reflectance of the POM-PTFE-Al film in the 4–25 μm waveband. Source data are provided as a Source Data file.

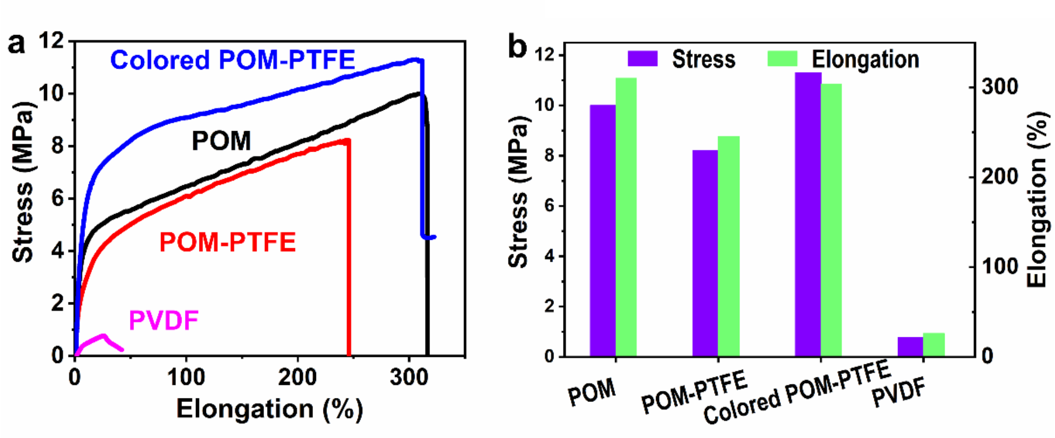

Supplementary Fig. 21. Mechanical strength test. a and b, Stress-strain curves (**a**) and the corresponding stress and elongation (**b**) of the dual-selective POM-PTFE, mono-selective POM, non-selective PVDF, and colored POM-PTFE (covered with red PE film, as shown in Figs. 5b and 5c). Source data are provided as a Source Data file.

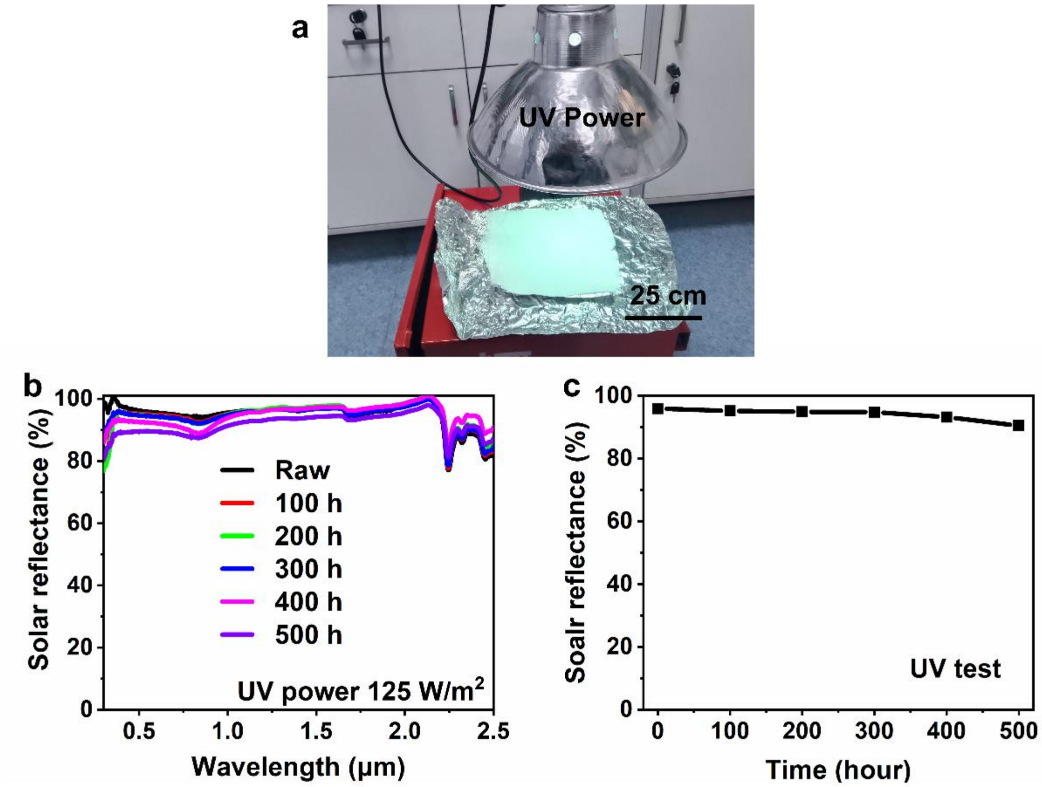

Supplementary Fig. 22. Intense UV exposure testings. a, Photograph of a UV power device. **b** and **c**, Spectral response (**b**, solar reflectance) and the average reflectance (**c**) of the dual-selective POM-PTFE film before and after UV testing.

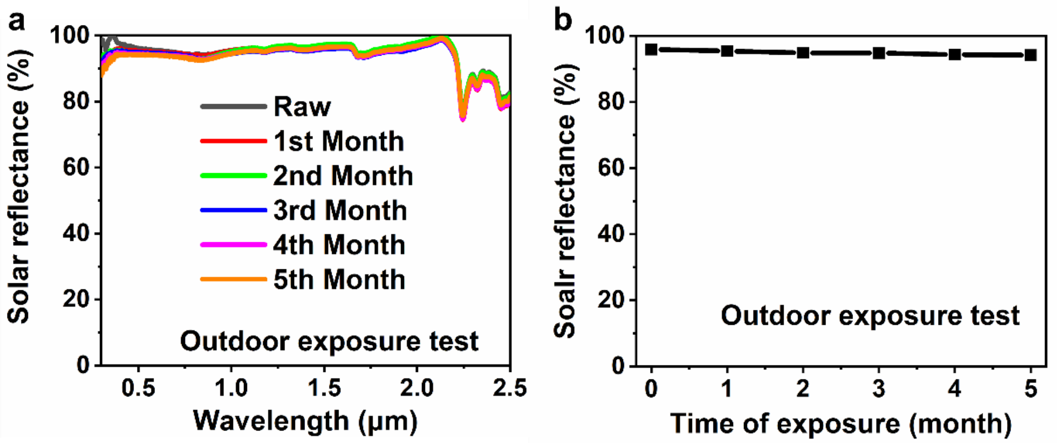

Supplementary Fig. 23. Outdoor exposure testings. a and **b**, Spectral response (**a**, solar reflectance) and the corresponding average reflectance (**b**) of the dual-selective POM-PTFE film before and after outdoor testing.

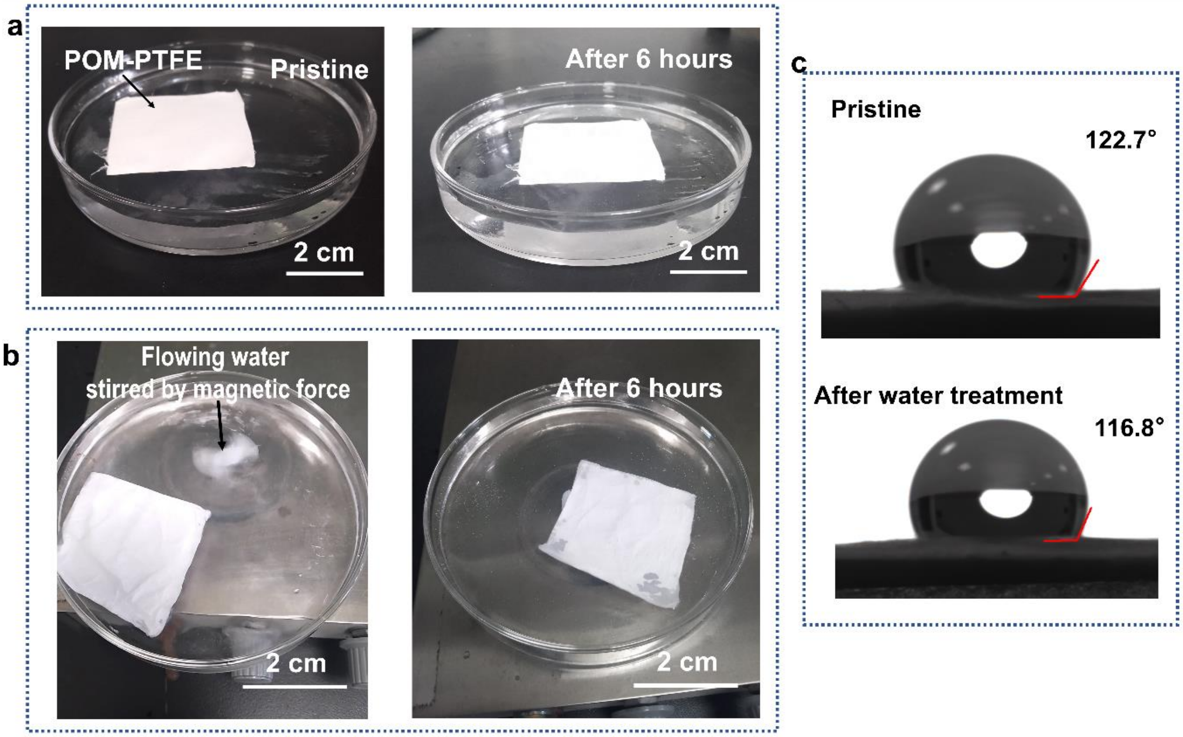

Supplementary Fig. 24. Water resistance test. a, Photograph of a dual-selective POM-PTFE film on non-flowing water before and after 6-hour water treatment. **b**, Photograph of a POM-PTFE film on flowing water (stirred by magnetic force) before and after 6-hour water treatment. **c**, Water contact angle of the dual-selective POM-PTFE film before and after 12-hour testing.

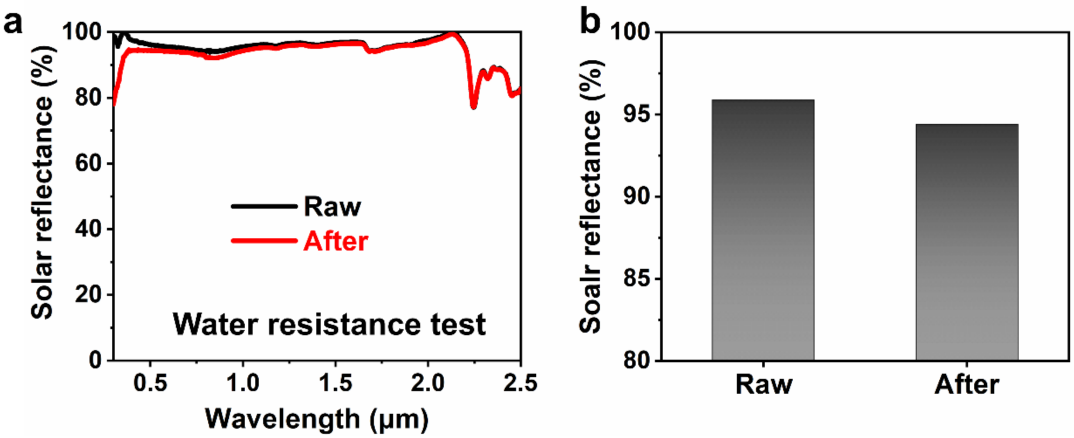

Supplementary Fig. 25. Water resistance test. Solar reflectance spectra (**a**) and the corresponding average solar reflectance (**b**) of a dual-selective POM-PTFE film before and after 12 h water treatment. Source data are provided as a Source Data file.

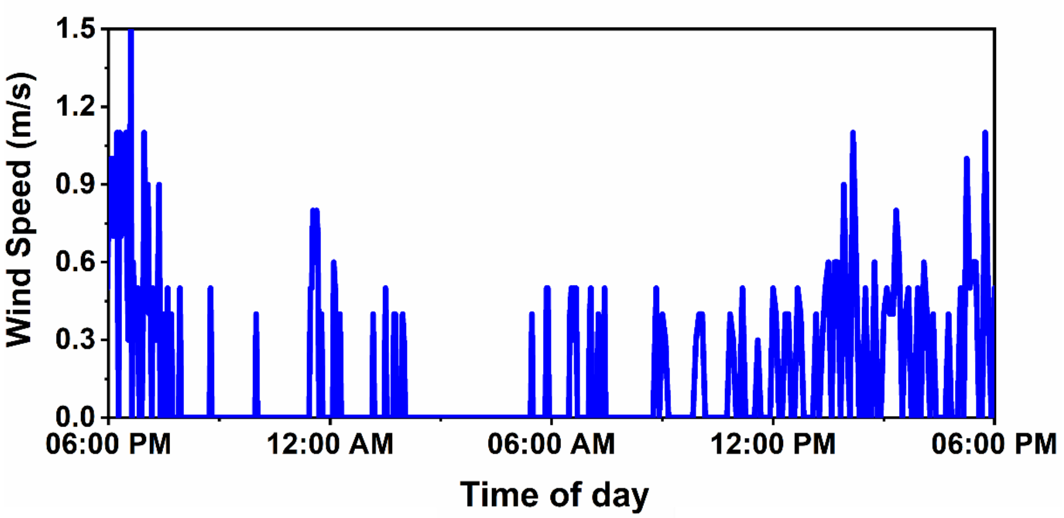

Supplementary Fig. 26. Wind speed during an outdoor thermal measurement (6 and 7 September 2022). Source data are provided as a Source Data file.

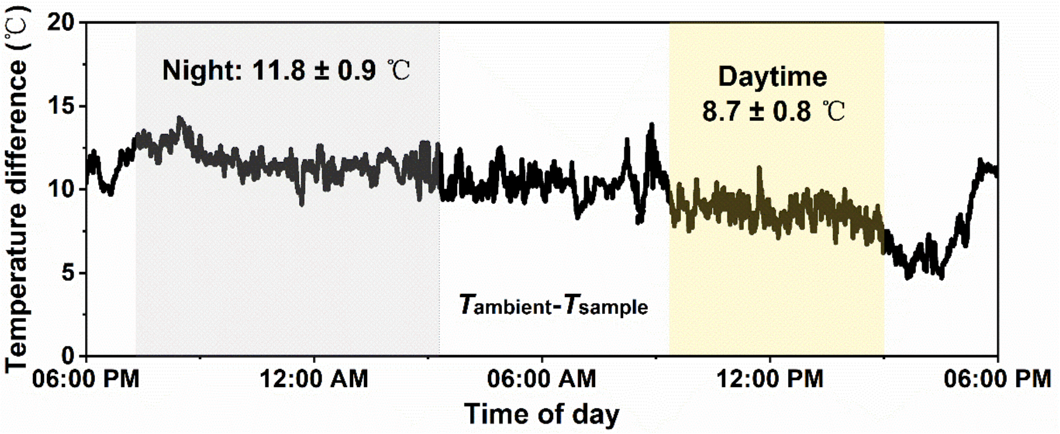

Supplementary Fig. 27. Temperature difference between ambient air and a dual-selective POM-PTFE film during an outdoor thermal measurement (6 and 7 September 2022). Source data are provided as a Source Data file.

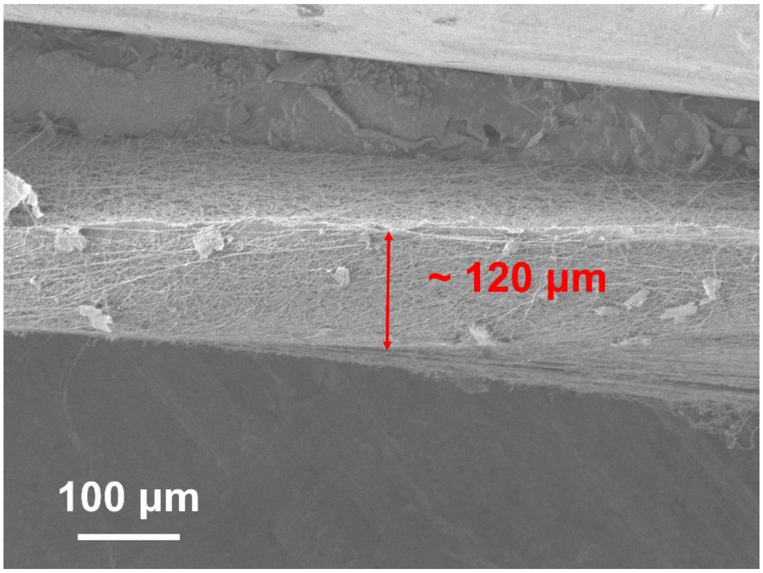

Supplementary Fig. 28. Cross-sectional SEM image of a mono-selective POM film. The thickness of the POM film is ~120 µm.

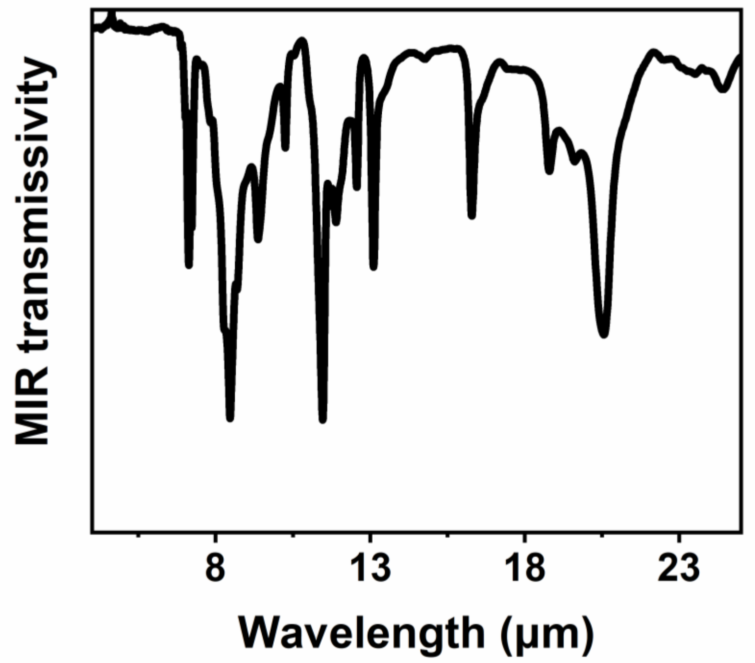

Supplementary Fig. 29. FTIR-ATR spectra of PVDF. The characteristic peaks are distributed over the entire MIR band, including the non-windowed bands within it.

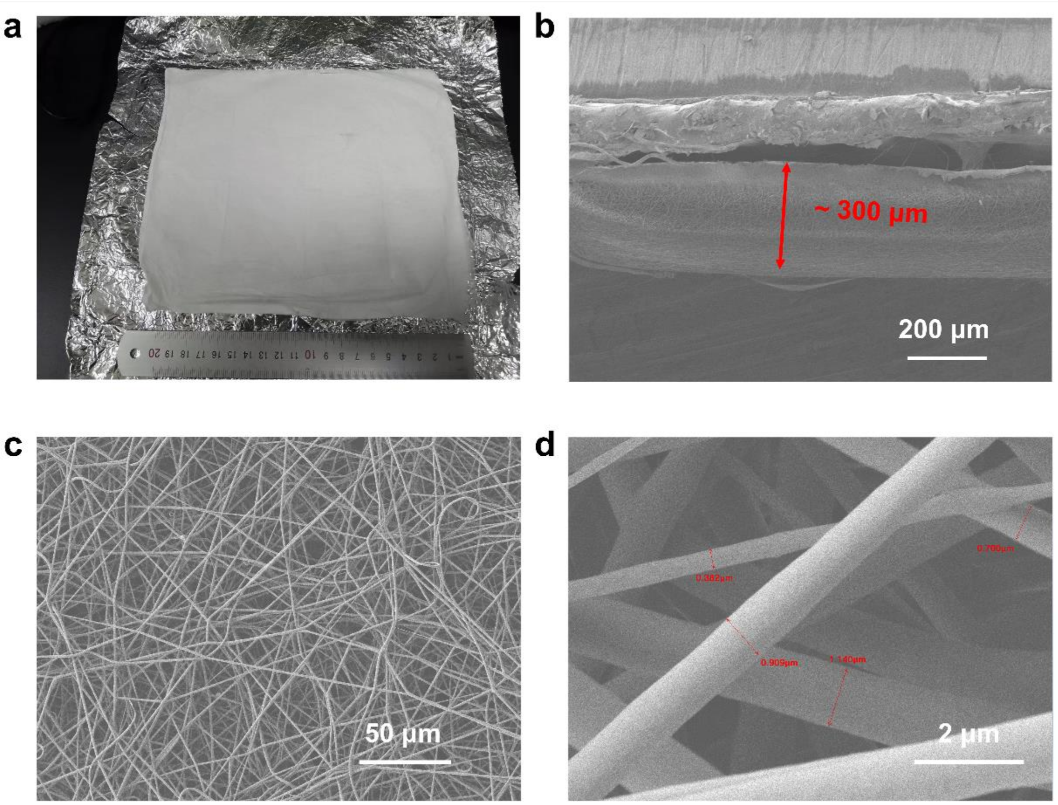

Supplementary Fig. 30. Images of the non-selective PVDF film. a, Digital image of a PVDF film. **b**, Cross-sectional SEM image of the PVDF film (~300 μm). **c** and **d**, SEM images of the PVDF nanofibers.

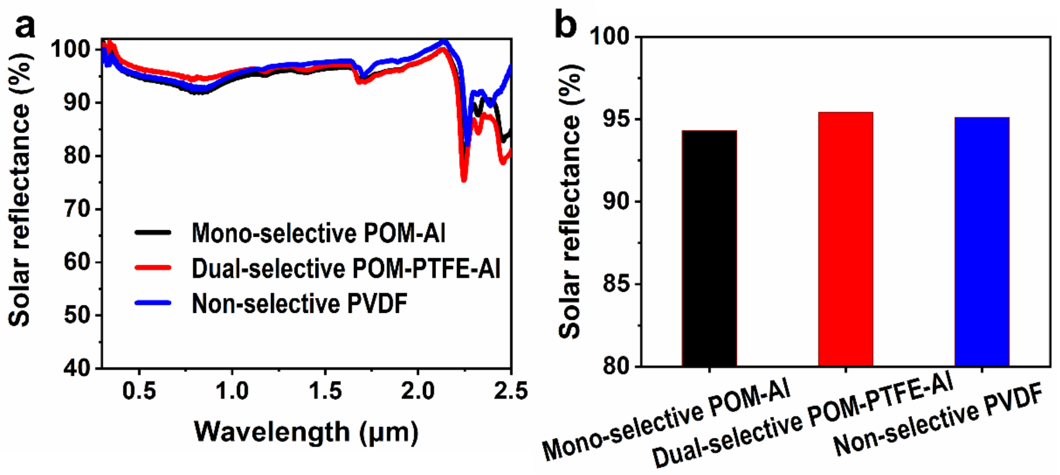

Supplementary Fig. 31. Comparison of the solar reflectance (0.3–2.5 μm) between dual-selective POM-PTFE-Al, mono-selective POM-Al, and non-selective PVDF films. Solar reflectance spectra (**a**) and the corresponding average solar reflectance (**b**). Source data are provided as a Source Data file.

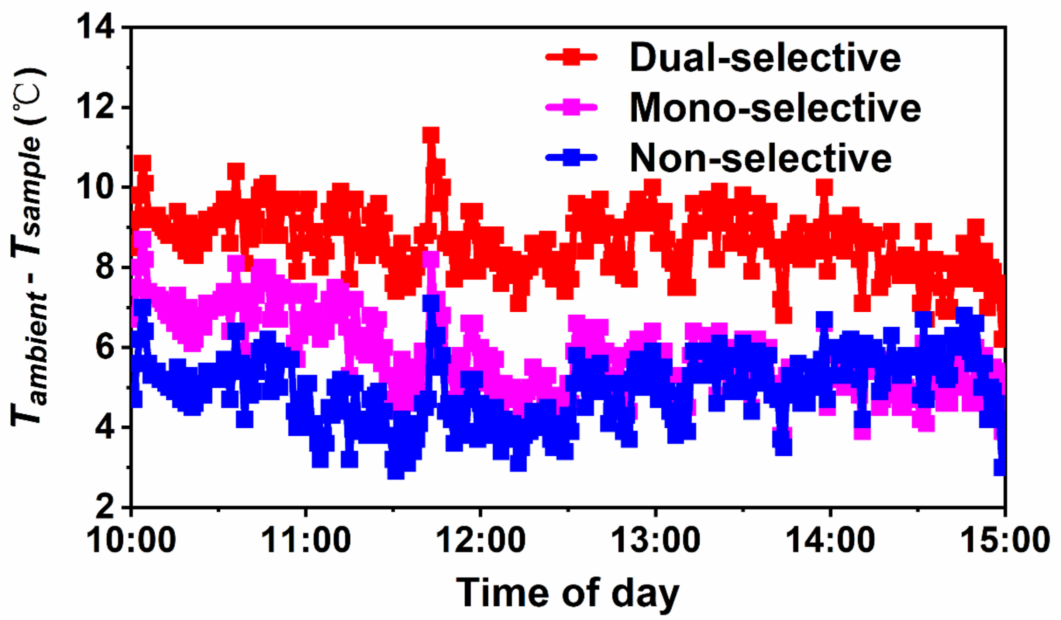

Supplementary Fig. 32. Temperature difference between the ambient air and three different samples (dual-selective POM-PTFE-Al, mono-selective POM-PTFE-Al, and non-selective PVDF films, respectively) during an outdoor thermal measurement. Source data are provided as a Source Data file.

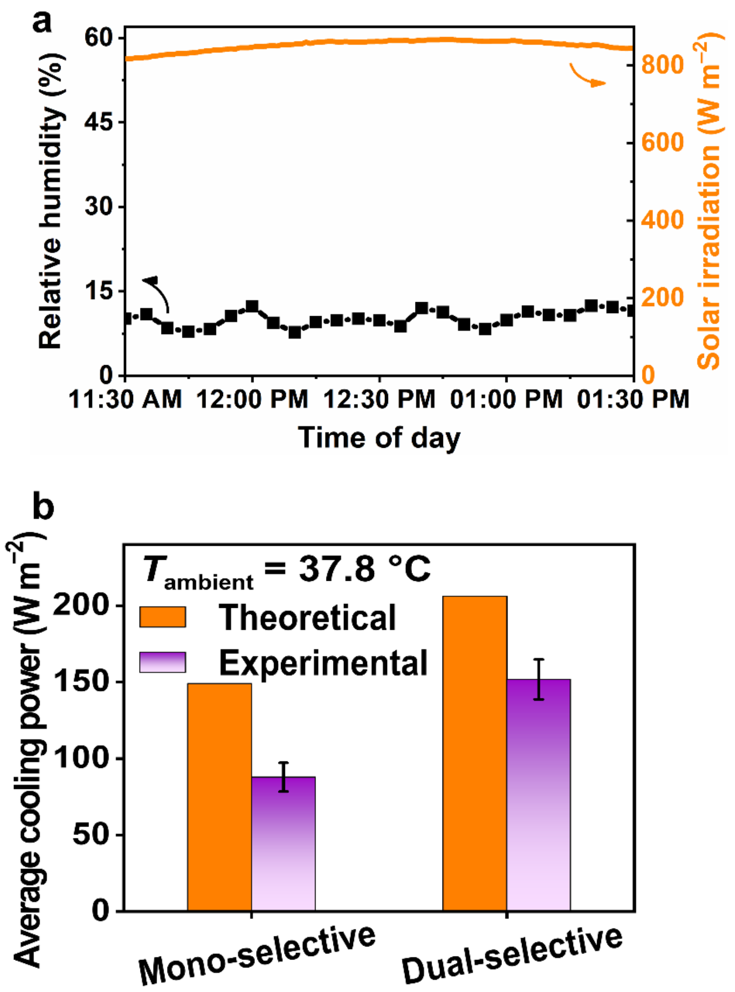

Supplementary Fig. 33. Cooling power measurements of the dual-selective emitter (in the Ulan Buh Desert, RH = ~10%, 7 September 2022). a, RH and solar irradiation during the thermal measurement from 11:30 a.m. to 01:30 p.m. **b**, Correlation between the simulated and the measured cooling powers, including the average cooling power of the dual-selective POM-PTFE-Al and mono-selective POM-Al from 11:30 a.m. to 01:30 p.m. and the theoretical cooling power. Error bars in **b** indicate measurement variations of the samples at different times and show the mean ± s.d. (n = 227 and 213 for the dual-selective and mono-selective samples, respectively). Source data are provided as a Source Data file.

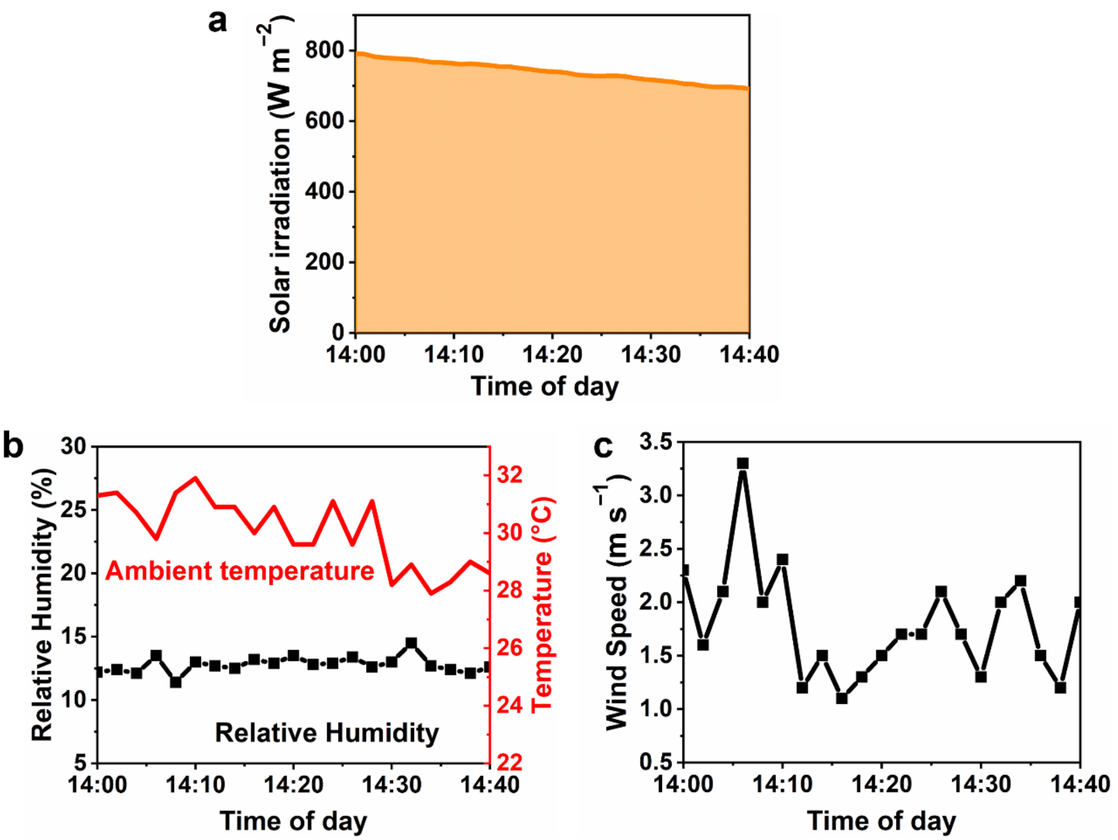

Supplementary Fig. 34. Ambient conditions of the cooling power measurements for the dual-selective and non-selective films (in the Ulan Buh Desert, 9 September 2022). a, Solar irradiation. b, Ambient air temperature and relative humidity. c, Wind speed.

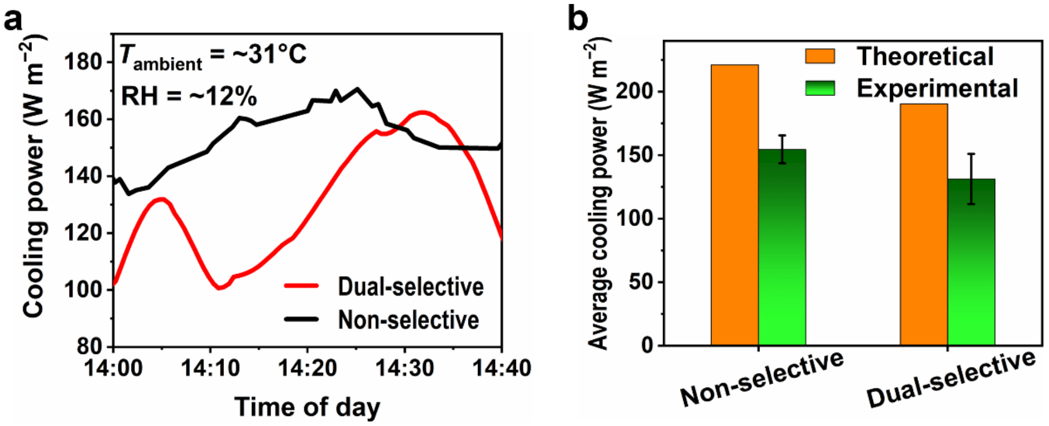

Supplementary Fig. 35. Cooling power measurements (in the Ulan Buh Desert, RH = ~12%, 9 September 2022). a, Real-time cooling power measurements of the dual-selective POM-PTFE-Al and non-selective PVDF from 02:00 a.m. to 02:50 p.m. **b**, Correlation between the simulated and the measured cooling powers, including the average cooling power of the dual-selective POM-PTFE-Al and non-selective PVDF and the corresponding theoretical cooling power. Error bars in **b** indicate measurement variations of the samples at different times and show the mean ± s.d. (n = 80). Source data are provided as a Source Data file.

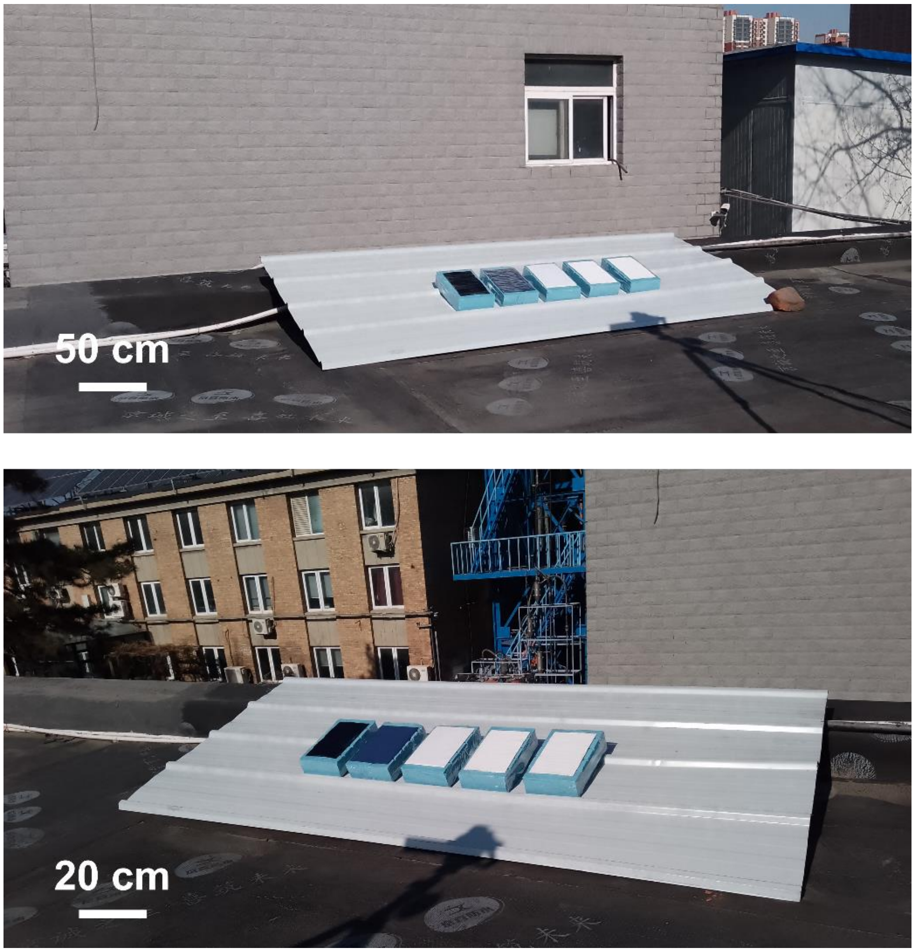

Supplementary Fig. 36. Photograph of several typical commercial roof materials, including a commercial color steel roof as a background (white), a commercial black asphalt, and 4 covered/coated black asphalts (an Al foil covered, a dual-selective film covered, a white paint-Al covered, and a white paint coated).

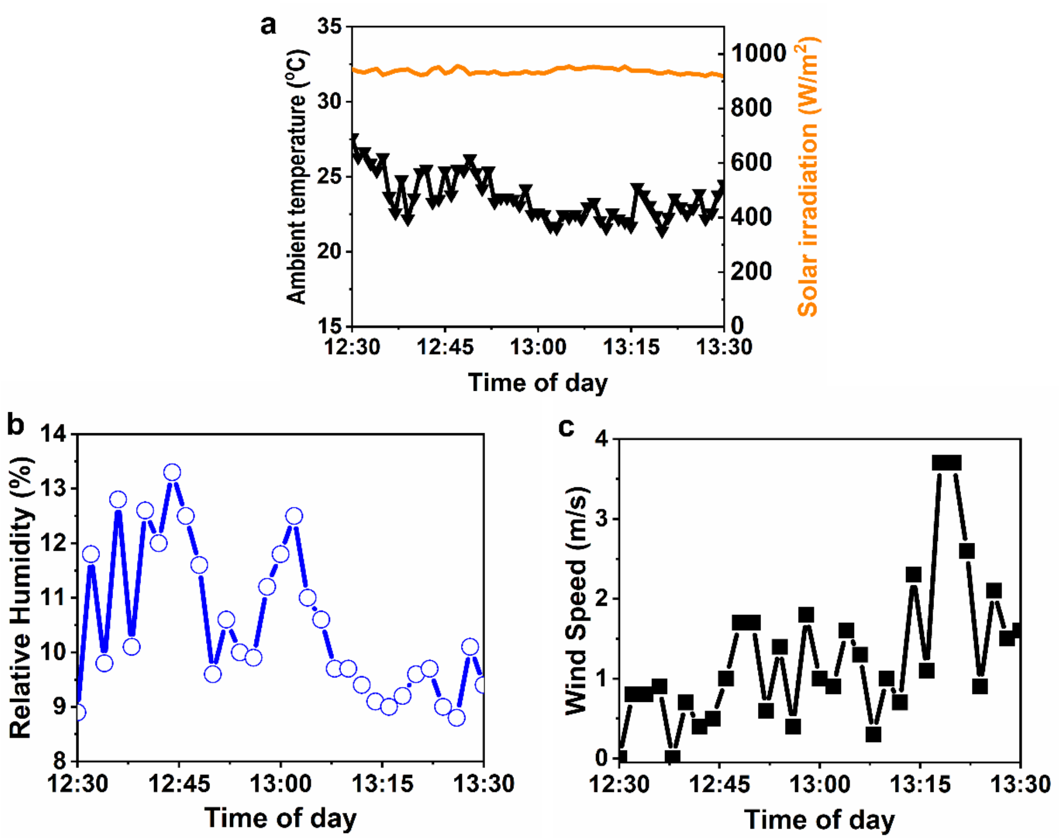

Supplementary Fig. 37. Ambient conditions of the outdoor IR measurement for the dual-selective PTFE-POM film (Beijing, China, 31 January 2023**). a**, Ambient air temperature and solar irradiation. **b**, Relative humidity. **c**, Wind speed.

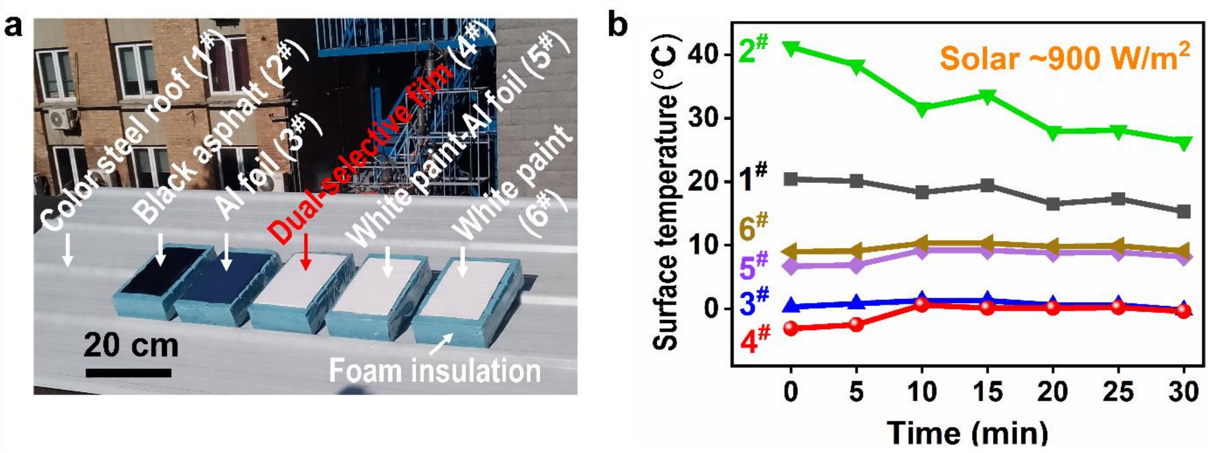

Supplementary Fig. 38. Outdoor cooling performance measurements of the dual-selective emitter (Beijing, China, 31 January 2023). a, photograph, and **b,** the corresponding real-time surface temperatures of the dual-selective emitter and the several typical commercial roofing materials, including color steel roof (white, background, marked as 1#), uncovered black asphalt (10×20 cm^2, marked as 2#), and covered black asphalt (Al foil-covered 3#, dual-selective film covered 4#, white paint-Al foil covered 5#, and white paint-coated 6#). It should be noted that the infrared tests are qualitative as the colors of an infrared image depend not only on the surface temperature but also on the MIR emittance of sample materials. In this test, the emissivity of each sample in the infrared images was not calibrated individually and it was assumed to have an emissivity of 0.9, which is obviously inappropriate for the ultra-low emissivity Al foil sample. Thus, the cooling performance between the Al foil and the dual-selective sample cannot be judged from the colors of their infrared images alone. Source data are provided as a Source Data file.

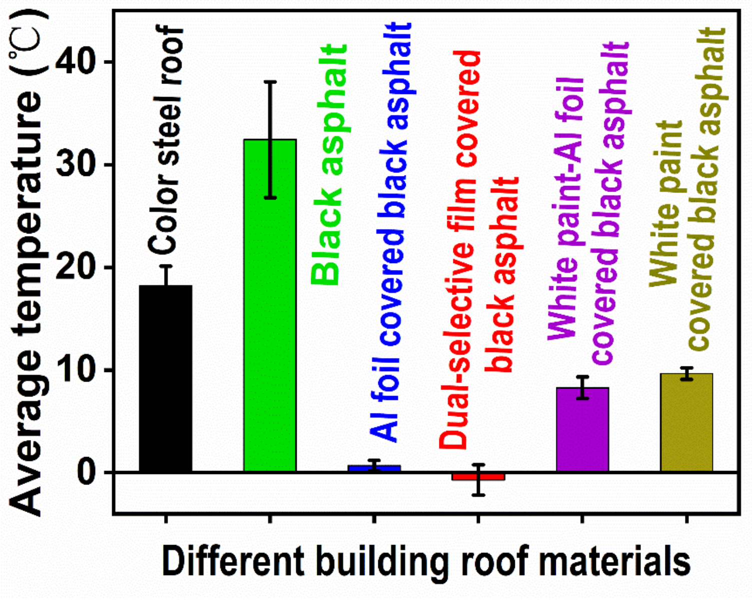

Supplementary Fig. 39. Average surface temperatures of different building roof materials. Error bars indicate measurement variations of the samples at different times and show the mean ± s.d. (n = 7).

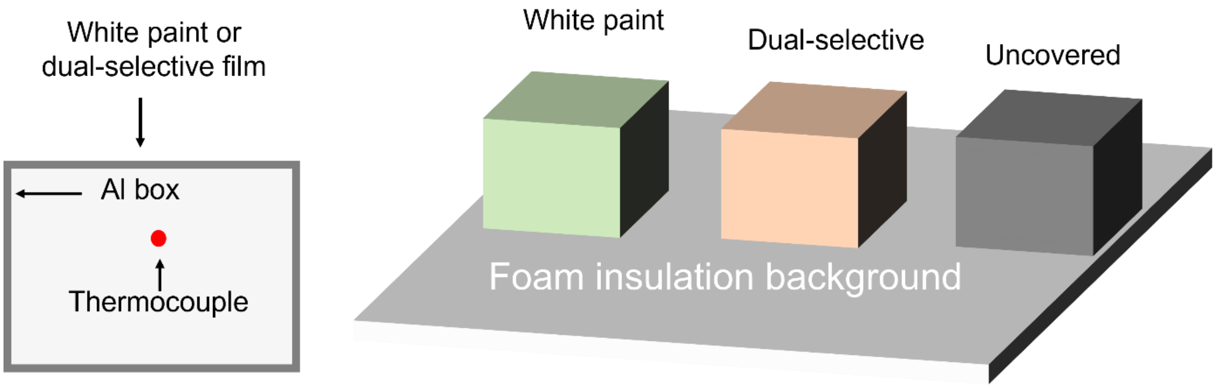

Supplementary Fig. 40. Schematics of the thermal measurements of the building model.

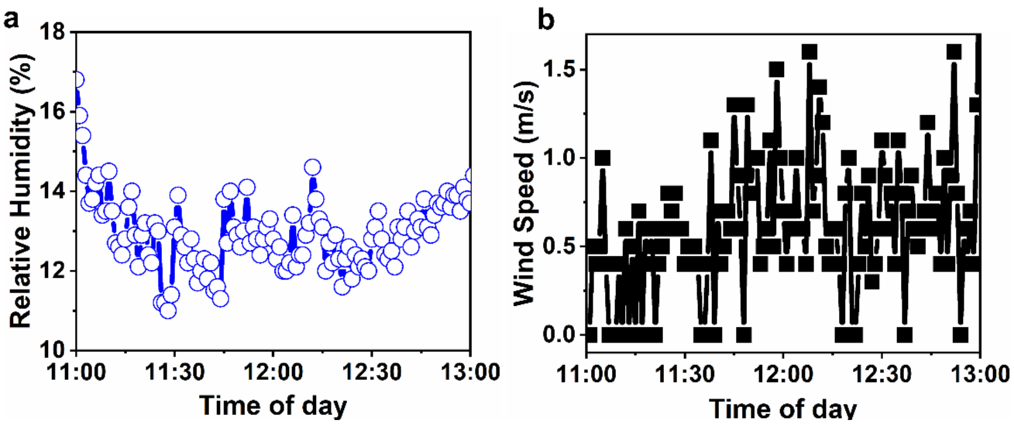

Supplementary Fig. 41. Ambient conditions of the temperature measurement of Al foil boxes (Beijing, China, 29 March 2023**). a**, Relative humidity. **b**, Wind speed.

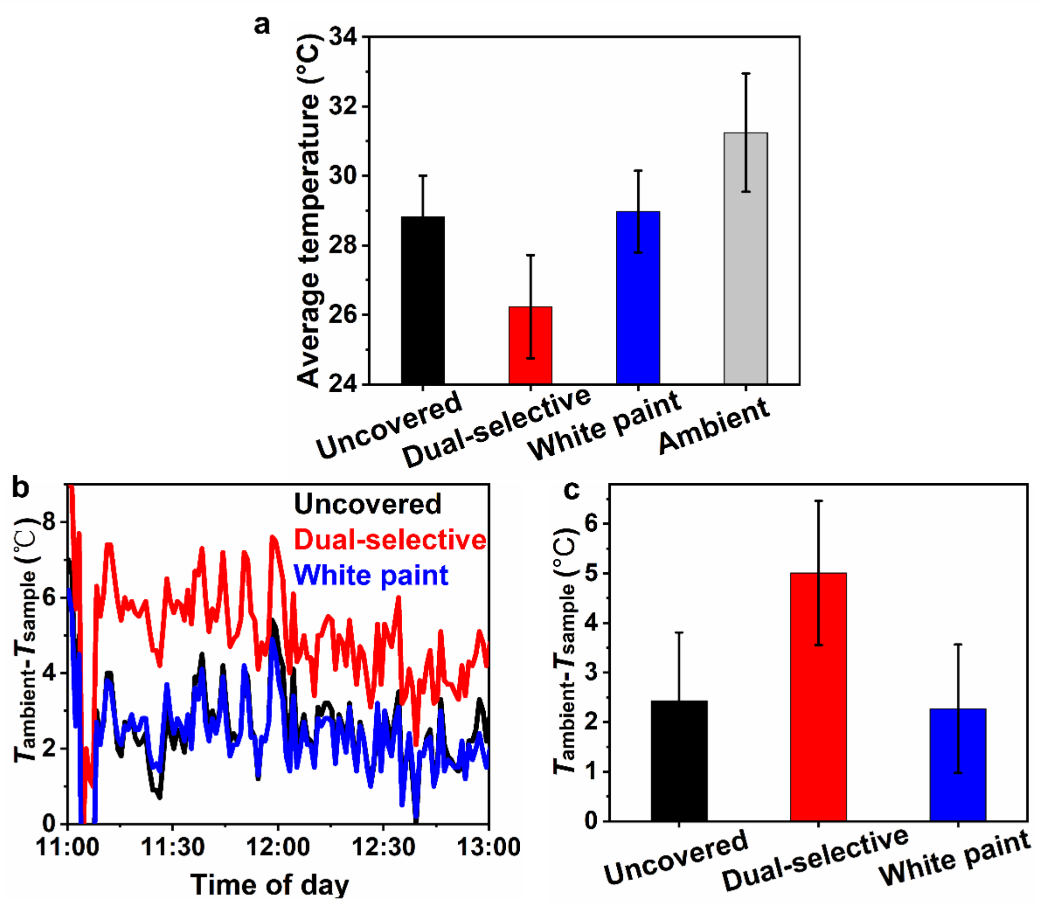

Supplementary Fig. 42. Temperature measurement of a dual-selective film-covered Al foil box. a, Average internal temperature of the uncovered, dual-selective film-covered, and white paint-covered Al foil boxes. The average ambient temperature is also included for comparison. **b and c**, Temperature difference (**b**) and the corresponding average values (**c**) between the ambient air and the interior of three Al foil boxes. Error bars in **a** and **c** indicate measurement variations of the samples at different times (measured at one-minute intervals from 11:30 a.m. to 13:00 a.m.) and show the mean ± s.d. (n = 91).

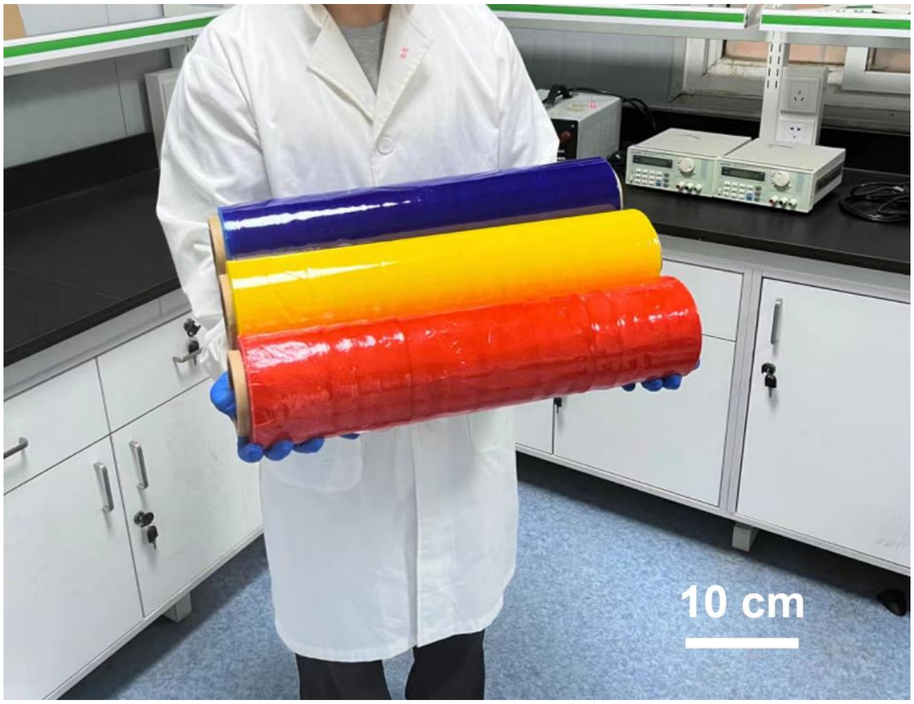

Supplementary Fig. 43. Digital image of commercial-colored PE films (red, yellow, and blue, respectively).

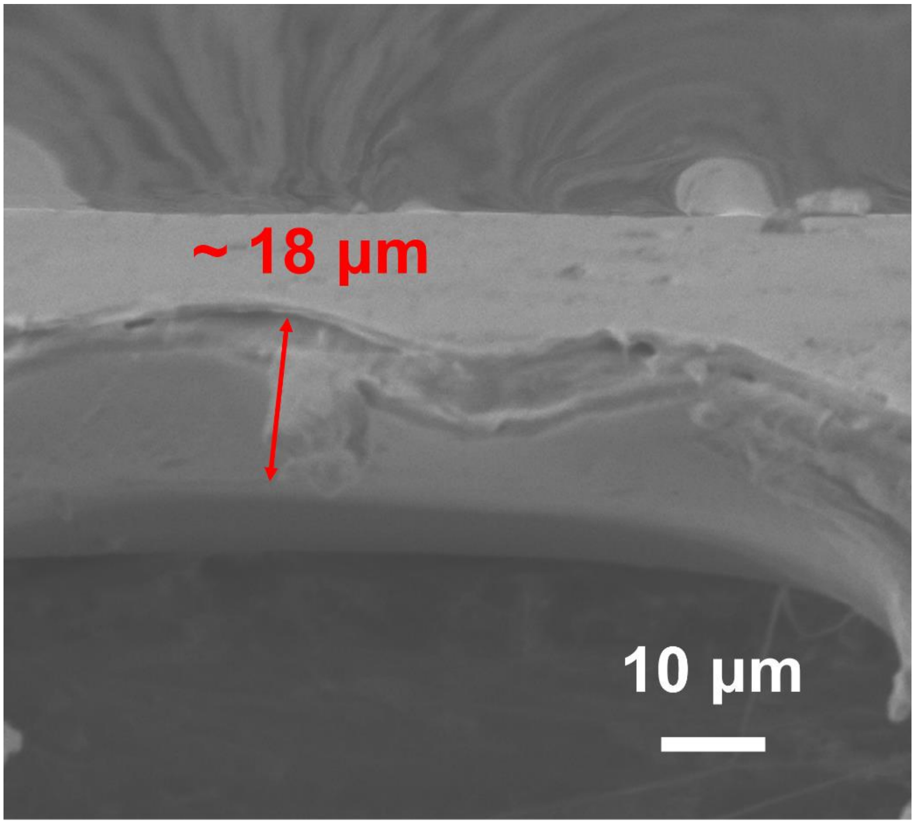

Supplementary Fig. 44. Cross-sectional SEM image of a commercial-colored PE film. The thickness of the colored PE film is ~18 μm.

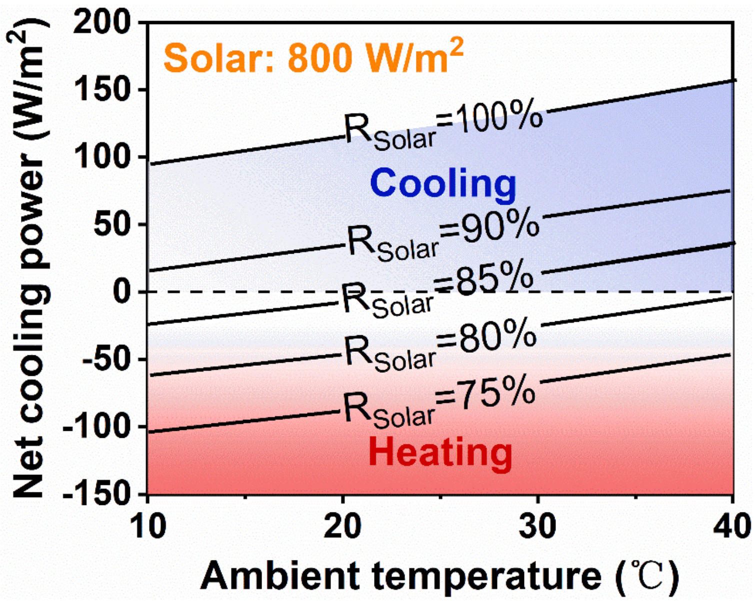

Supplementary Fig. 45. Theoretical cooling power of a mono-selective thermal emitter as a function of ambient temperature for different R_{solar}.

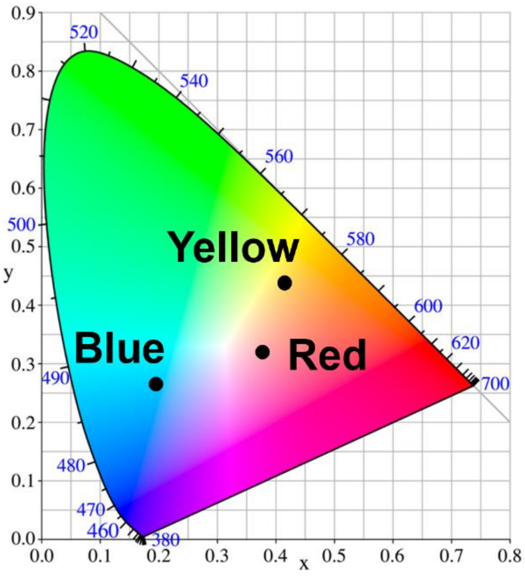

Supplementary Fig. 46. Commission Internationale de l’Eclairage (CIE) chromaticity coordinates of red/yellow/blue dual-selective films.

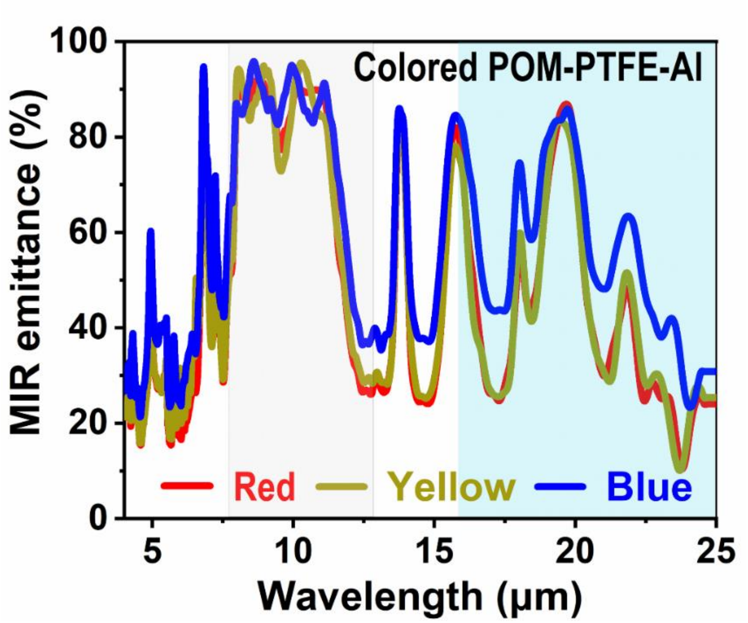

Supplementary Fig. 47. MIR emittance of three different colored POM-PTFE films (4–25 μm).

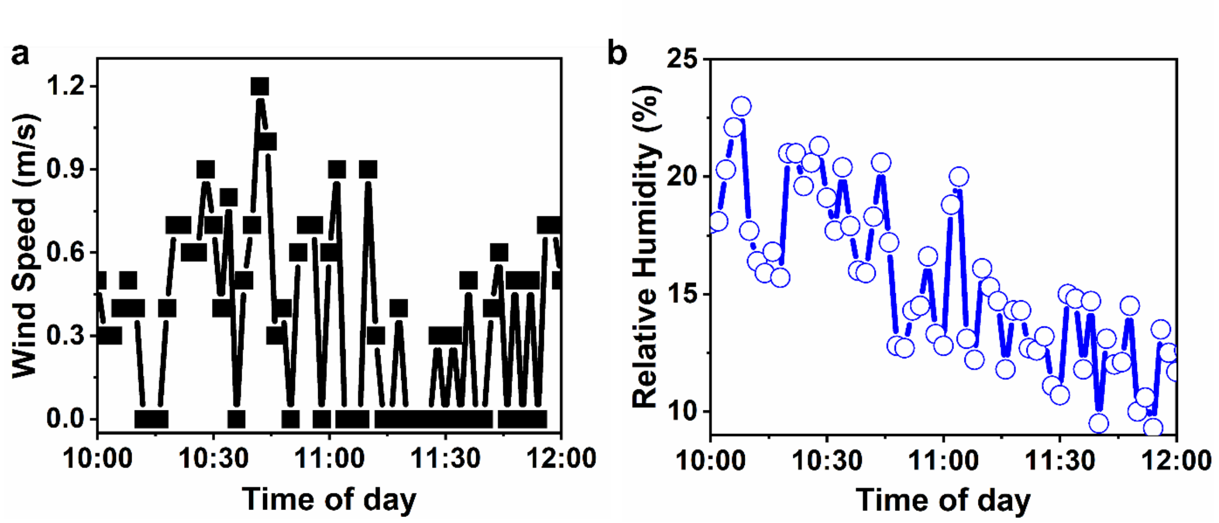

Supplementary Fig. 48. Ambient conditions of an outdoor thermal measurement for the colored dual-selective PTFE-POM films. a, Wind speed. **b**, Relative humidity.

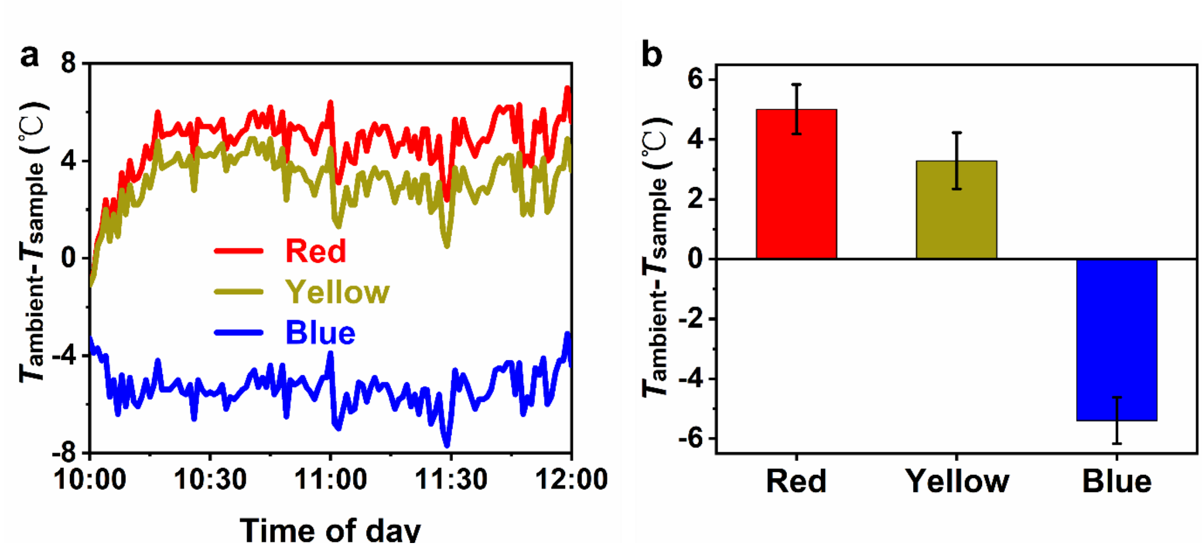

Supplementary Fig. 49. Outdoor thermal measurement for the colored dual-selective POM-PTFE films. **a**, Temperature difference between ambient air and three colored samples (red, yellow, and blue, respectively). **b**, Average temperature difference of the three colored samples. Error bars in **b** and **c** indicate measurement variations of the samples at different times (measured at one-minute intervals from 10:30 a.m. to 12:00 a.m.) and show the mean ± s.d. (n = 91). Source data are provided as a Source Data file.

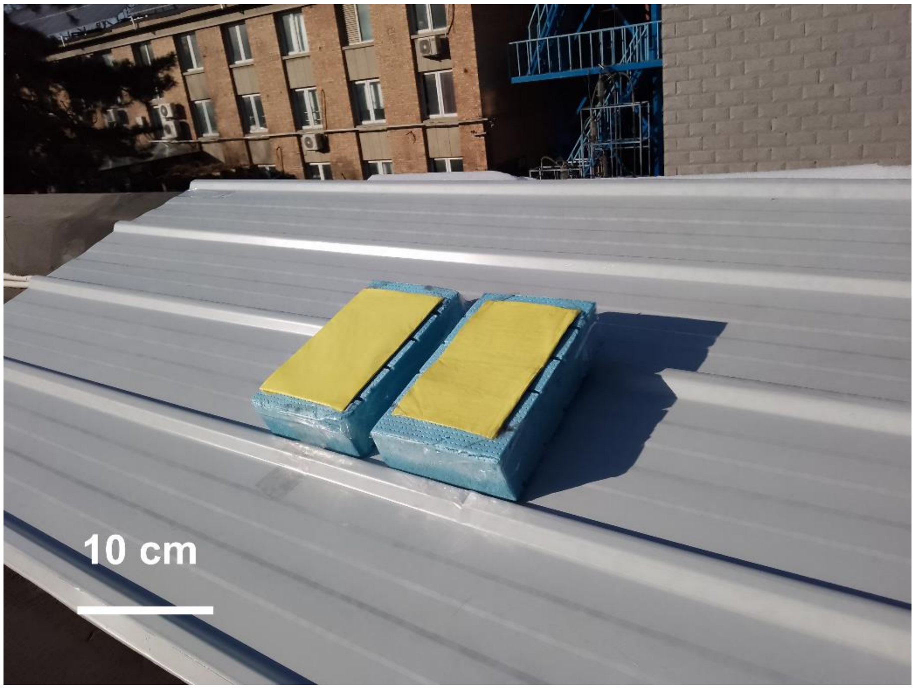

Supplementary Fig. 50. Photograph of a yellow dual-selective film covered asphalt (left) and a yellow PE film covered white painted asphalt.

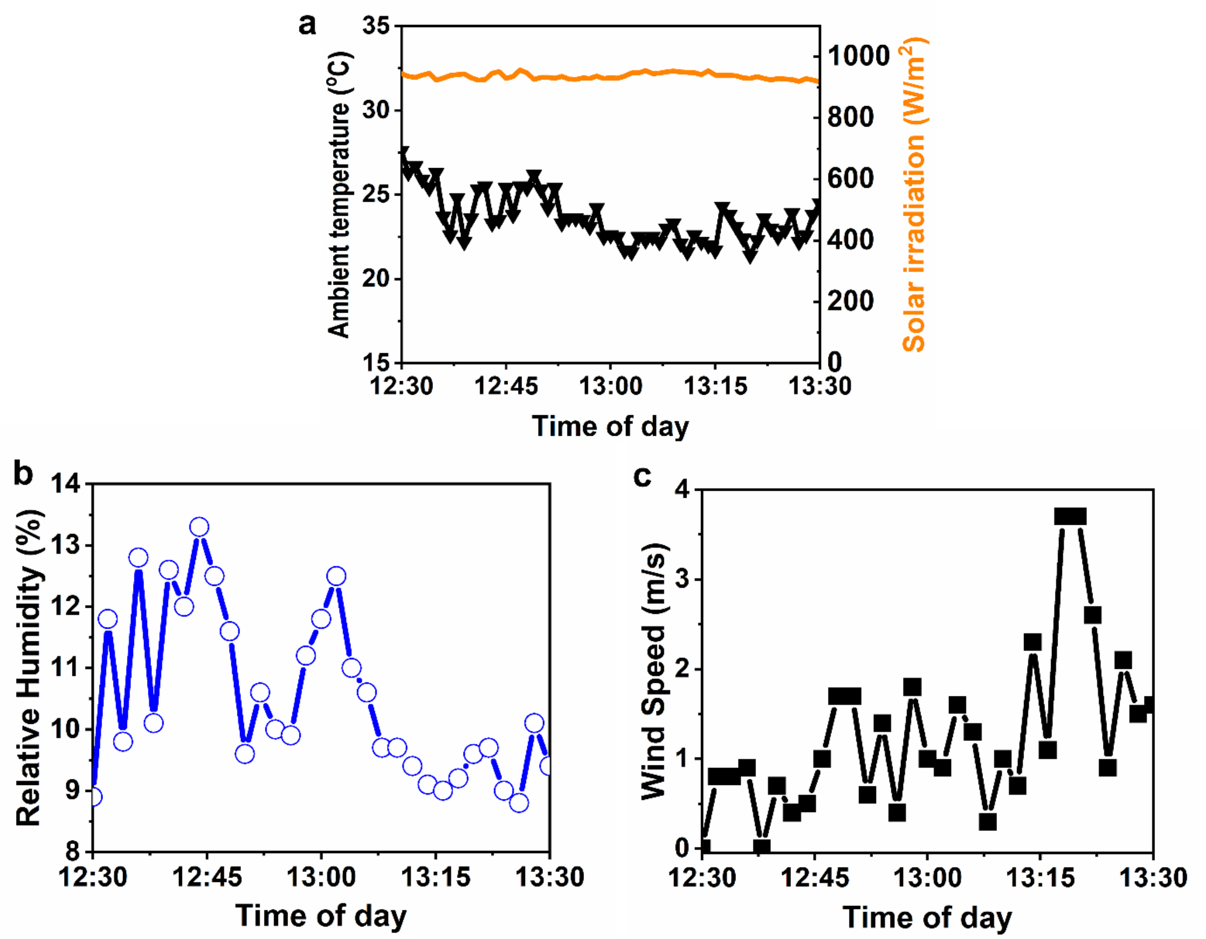

Supplementary Fig. 51. Ambient conditions of the outdoor IR measurement for a yellow dual-selective PTFE-POM film (Beijing, China, 31 January 2023**). a**, Ambient air temperature and solar irradiation. **b**, Relative humidity. **c**, Wind speed.

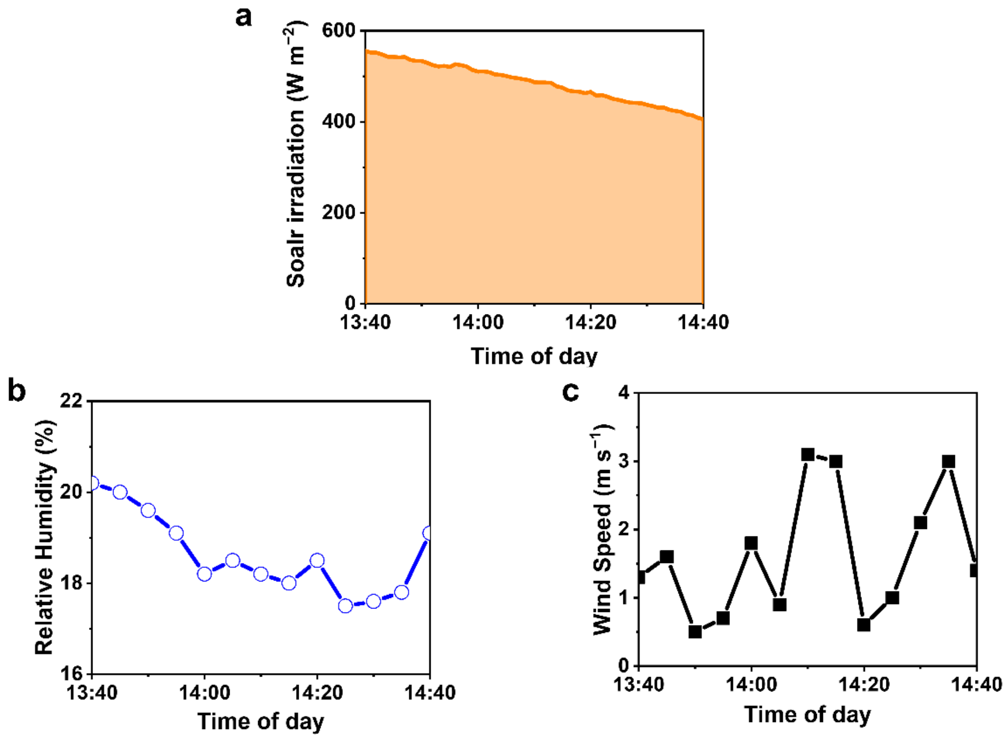

Supplementary Fig. 52. Ambient conditions of the outdoor IR measurement for a blue dual-selective PTFE-POM-Al film (Beijing, China, 8 October 2023**). a**, Solar irradiation. **b**, Relative humidity. **c**, Wind speed.

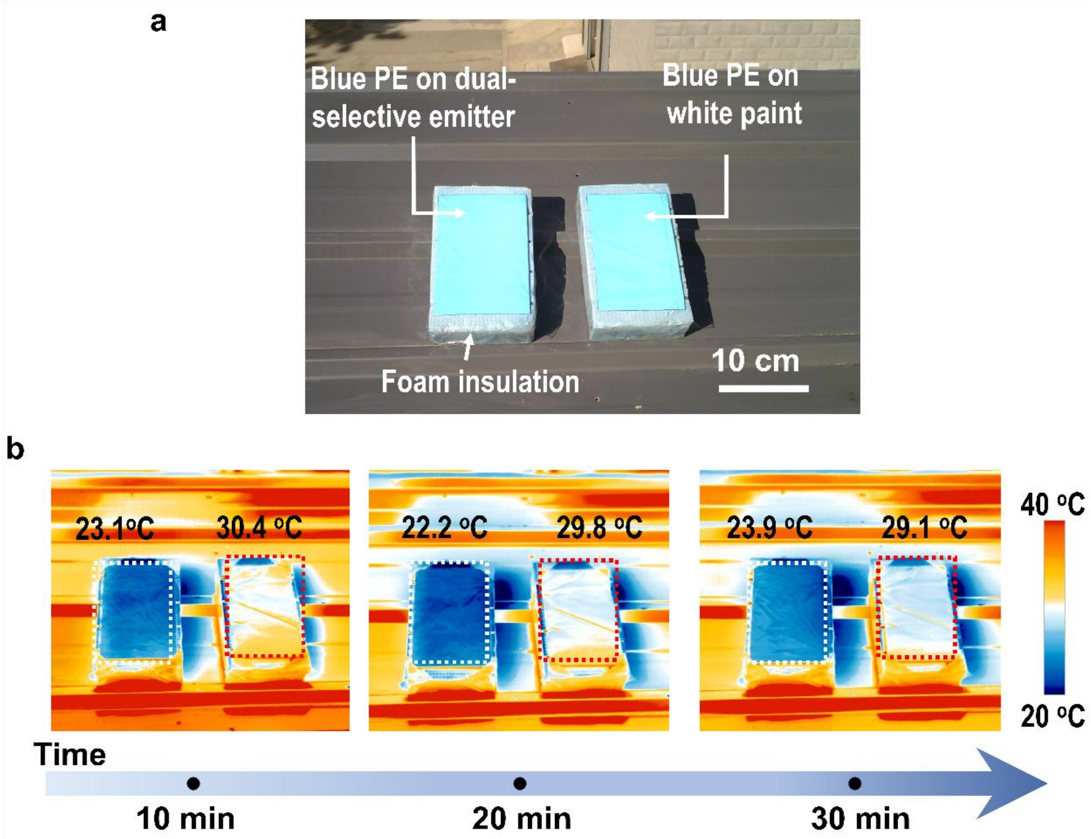

Supplementary Fig. 53. Actual outdoor cooling performance test for the blue dual-selective thermal emitter covered asphalt in Beijing, China (RH = ~18%, 8 October 2023) compared to the blue PE film covered white painted asphalt, including their photograph (a) and IR images (b).

Supplementary Table 1. Theoretical limit of the subambient temperature reduction at different non-radiative heat transfer coefficient h (0–6 W m^{-2} K^{-1}) for the three different radiative coolers

h (W m^{-2} K^{-1})	Theoretical subambient temperature reduction (ºC)		
	Dual-selective	Mono-selective	Non-selective
0	75.8	90.0	47.6
1	46.9	42.3	36.0
2	35.4	29.9	29.5
3	28.7	23.4	25.1
4	24.2	19.3	21.0
5	21.0	16.4	19.5
6	18.6	14.3	17.6

Supplementary Table 2. Characteristic peaks and their functional group vibrations in the FTIR-ATR spectrum of POM and PTFE.

Wavenumber (cm^{-1})	Wavelength (μm)	Mode of vibration
1234.6	8.1	Stretching vibration of -CH2
1201.9, 1149.4	8.3, 8.7	Stretching vibration of F-C-F
1087.0, 934.6, 900.9	9.2, 10.7, 11.1	Stretching vibration of C-O-C
628.9	15.9	Formation vibration of O-C-O
636.9, 624.0	15.7, 16.0	Bending vibration of C-F
554.0, 505.0	18.1, 19.8	Formation vibration of C-F
456.6	21.9	Skeletal Vibration of POM

Supplementary Table 3. Tensile strength of the dual-selective POM-PTFE, mono-selective POM, Non-selective PVDF, and colored POM-PTFE (red) films.

Sample	Stress (Mpa)	Elongation (%)
POM-PTFE	8.2	245.5
Colored POM-PTFE (red)	11.3	304.0
PVDF	0.77	26.2
POM	10.0	310.1

Supplementary references

1 Chen, Z., Zhu, L., Raman, A. & Fan, S. Radiative cooling to deep sub-freezing temperatures through a 24-h day-night cycle. *Nat. Commun.* **7**, 13729 (2016).

2 Lord, S. D. in *In NASA Technical Memorandum.* 103957 (NASA).

3 Zhu, R. K. *et al.* Plasmon-Enhanced Infrared Emission Approaching the Theoretical Limit of Radiative Cooling Ability. *Nano Lett.* **20**, 6974–6980 (2020).

4 Kam, Z. Absorption and Scattering of Light by Small Particles - Bohren,C, Huffman,Dr. *Nature* **306**, 625–625 (1983).

5 Doremus, R. H. Scattering and Absorption of Light by Small Metallic Particles in a Thin Film. *J. Colloid Interf. Sci.* **27**, 412 (1968).

6 Chandrasekhar, S. *Radiative Transfer* (Courier Corporation, 2013).

7 Huang, Z. F. & Ruan, X. L. Nanoparticle embedded double-layer coating for daytime radiative cooling. *Int. J. Heat Mass Tran.* **104**, 890–896 (2017).

8 Zeng, S. *et al.* Hierarchical-morphology metafabric for scalable passive daytime radiative cooling. *Science* **373**, 692–696 (2021).

9 Cooke, A. Infrared-Spectra of Polyoxymethylene Grains. *Astrophys. Space Sci.* **39**, L13–L18 (1976).

10 Whittet, D. C. B., Dayawansa, I. J., Dickinson, P. M., Marsden, J. P. & Thomas, B. Optical-Constants of Polyoxymethylene. *Mon. Not. R. Astron. Soc.* **175**, 197–207 (1976).

11 Folks, W., Pandey, S. & Boreman, G. Refractive Index at THz Frequencies of Various Plastics. *Optics InfoBase Conference Papers*, (2007).

12 Lloyd, M. Thermal Imaging-Systems Principles. *J. Opt. Soc. Am.* **66**, 389–389 (1976).

13 Wu, X. *et al.* An all-weather radiative human body cooling textile. *Nat. Sustain.*, **6**, 1446–

1454 (2023).

14 Bognitzki, M. *et al.* Nanostructured Fibers via Electrospinning. *Adv. Mater.* **13**, 70–72 (2001).

15 Xue, J. J., Wu, T., Dai, Y. Q. & Xia, Y. N. Electrospinning and Electrospun Nanofibers: Methods, Materials, and Applications. *Chem. Rev.* **119**, 5298–5415 (2019).

16 Doshi, J. & Reneker, D. H. Electrospinning Process and Applications of Electrospun Fibers. *J. Electrostat.* **35**, 151–160 (1995).

17 Al-Abduljabbar, A. & Farooq, I. Electrospun Polymer Nanofibers: Processing, Properties, and Applications. *Polymers-Basel* **15**, 65 (2023).

18 Lu, J. W. *et al.* High-elongation fiber mats by electrospinning of polyoxymethylene. *Macromolecules* **41**, 3762–3764 (2008).

19 Chen, F. *et al.* Superdurable and fire-retardant structural coloration of carbon nanotubes. *Sci. Adv.* **8**, eabn5882 (2022).

20 Mandal, J. *et al.* Hierarchically porous polymer coatings for highly efficient passive daytime radiative cooling. *Science* **362**, 315–318 (2018).

21 Aili, A. *et al.* Selection of polymers with functional groups for daytime radiative cooling. *Mater. Today Phys.* **10**, 100127 (2019).

22 Song, Y. N., Lei, M. Q., Lei, J. & Li, Z. M. Spectrally selective polyvinylidene fluoride textile for passive human body cooling. *Mater. Today Energy* **18**, 100504 (2020).
