# Supplementary material for: A dual-selective thermal emitter with enhanced subambient radiative cooling performance
Source: Nat Commun. 2024 Jan 27;15:815. doi: 10.1038/s41467-024-45095-4 (PMC10821923; doi:10.1038/s41467-024-45095-4)
Supplement: Supplementary file 3 — Description of Additional Supplementary Files [file 41467_2024_45095_MOESM3_ESM.pdf]

Description of Additional Supplementary Files

Supplementary Movie Legends

Supplementary Movie 1. The process of roll-to-roll electrospinning POM-PTFE electrospun film.

Supplementary Movie 2. Water resistance test video of the POM-PTFE based dual-selective thermal emitter (4×4 cm2).

Supplementary Movie 3. Infrared video of the dual-selective thermal emitter in an arid outdoor environment (RH = ~10%, 31 January 2023) of Beijing, China (40°0’33” N, 116°20’0.6” E). Several typical commercial roofing materials were used for comparison, including color steel roof (white, background), uncovered black asphalt (10×20 cm2), and covered black asphalt (Al foil-covered, white paint-Al foil covered, and white paint-coated).

Supplementary Movie 4. Infrared video of the yellow dual-selective thermal emitter in an arid environment in Beijing (RH = ~10%, 31 January 2023). The yellow PE film covered white painted asphalt was used for comparison.

Supplementary Movie 5. Infrared video of the blue dual-selective thermal emitter in an arid environment in Beijing (RH = ~18%, 8 October 2023). The blue PE film covered white painted asphalt was used for comparison.
