# Supplementary material for: A dual-selective thermal emitter with enhanced subambient radiative cooling performance
Source: Nat Commun. 2024 Jan 27;15:815. doi: 10.1038/s41467-024-45095-4 (PMC10821923; doi:10.1038/s41467-024-45095-4)
Supplement: Supplementary file 9 — Reporting Summary [file 41467_2024_45095_MOESM9_ESM.pdf]

nature portfolio

Corresponding author(s): Rufan Zhang

Last updated by author(s): Dec 6, 2023

Reporting Summary

Nature Portfolio wishes to improve the reproducibility of the work that we publish. This form provides structure for consistency and transparency in reporting. For further information on Nature Portfolio policies, see our Editorial Policies and the Editorial Policy Checklist.

Statistics

For all statistical analyses, confirm that the following items are present in the figure legend, table legend, main text, or Methods section.

n/a	Confirmed	
☐	☒	The exact sample size (*n*) for each experimental group/condition, given as a discrete number and unit of measurement
☒	☐	A statement on whether measurements were taken from distinct samples or whether the same sample was measured repeatedly
☒	☐	The statistical test(s) used AND whether they are one- or two-sided<br>*Only common tests should be described solely by name; describe more complex techniques in the Methods section.*
☒	☐	A description of all covariates tested
☒	☐	A description of any assumptions or corrections, such as tests of normality and adjustment for multiple comparisons
☐	☒	A full description of the statistical parameters including central tendency (e.g. means) or other basic estimates (e.g. regression coefficient) AND variation (e.g. standard deviation) or associated estimates of uncertainty (e.g. confidence intervals)
☒	☐	For null hypothesis testing, the test statistic (e.g. *F*, *t*, *r*) with confidence intervals, effect sizes, degrees of freedom and *P* value noted<br>*Give P values as exact values whenever suitable.*
☒	☐	For Bayesian analysis, information on the choice of priors and Markov chain Monte Carlo settings
☒	☐	For hierarchical and complex designs, identification of the appropriate level for tests and full reporting of outcomes
☒	☐	Estimates of effect sizes (e.g. Cohen's *d*, Pearson's *r*), indicating how they were calculated

Our web collection on statistics for biologists contains articles on many of the points above.

Software and code

Policy information about availability of computer code

Data collection	No software was used
Data analysis	No software was used

For manuscripts utilizing custom algorithms or software that are central to the research but not yet described in published literature, software must be made available to editors and reviewers. We strongly encourage code deposition in a community repository (e.g. GitHub). See the Nature Portfolio guidelines for submitting code & software for further information.

Data

Policy information about availability of data

All manuscripts must include a data availability statement. This statement should provide the following information, where applicable:

- Accession codes, unique identifiers, or web links for publicly available datasets
- A description of any restrictions on data availability
- For clinical datasets or third party data, please ensure that the statement adheres to our policy

The data that support the findings of this paper are available from the corresponding authors upon reasonable request. Correspondence and requests for materials should be addressed to R.Z. Source data are provided with this paper.

April 2023

Research involving human participants, their data, or biological material

Policy information about studies with human participants or human data. See also policy information about sex, gender (identity/presentation), and sexual orientation and race, ethnicity and racism.

Reporting on sex and gender	We don't have related issues
Reporting on race, ethnicity, or other socially relevant groupings	We don't have related issues
Population characteristics	We don't have related issues
Recruitment	We don't have related issues
Ethics oversight	We don't have related issues

Note that full information on the approval of the study protocol must also be provided in the manuscript.

Field-specific reporting

Please select the one below that is the best fit for your research. If you are not sure, read the appropriate sections before making your selection.

☐ Life sciences ☐ Behavioural & social sciences ☒ Ecological, evolutionary & environmental sciences

For a reference copy of the document with all sections, see nature.com/documents/nr-reporting-summary-flat.pdf

Ecological, evolutionary & environmental sciences study design

All studies must disclose on these points even when the disclosure is negative.

Study description	Here, a dual-selective polymer-metal thermal emitter was developed, which exhibited selective emission in both atmospheric transparent windows (8–13 µm and 16–25 µm) and reflection in the remaining mid-infrared and solar wavebands. As a result, the dual-selective thermal emitter exhibited an ultrahigh subambient daytime cooling performance, surpassing existing typical thermal emitters and commercial counterparts.
Research sample	We don't have related issues.
Sampling strategy	We don't have related issues.
Data collection	The data used for statistics in the article were collected by a contact K-type thermocouple pyrometer in the same locations.
Timing and spatial scale	The data used for the statistics were obtained in the same location, specifically a continuous test in a dry environment for 40 min to 24 hours, with data recorded every minute. The test times were sunny outdoor in the Ulan Buh desert, Inner Mongolia, China,(6,7 and 9 September 2022) and in Beijing, China (31 January 2023, 29 March 2023, and 8 October 2023).
Data exclusions	Data were not excluded from analysis
Reproducibility	All thermal measurement results in the article are repeatable when measured under similar outdoor conditions (such as ambient temperature, relative humidity, weather condition, solar irradiation and wind speed).
Randomization	We calculated the results for all samples without randomness.
Blinding	The data used for statistics in the article were measured by calibrated thermocouples in the same environment and there is no blinding issue.

Did the study involve field work? ☒ Yes ☐ No

Field work, collection and transport

Field conditions	Ulan Buh desert, Inner Mongolia, China,(from 6 to 7, September 2022): with a ambient temperature of 15~40 °C, a relative humidity of 5%-50%, and a top solar radiation of ~850 W m-2;<br>Ulan Buh desert, Inner Mongolia, China,(9 September 2022): with a ambient temperature of ~31 °C, a relative humidity of 12%, and a solar radiation of 600~800 W m-2;<br>Beijing, China (31 January 2023): with a ambient temperature of ~15 °C, a relative humidity of 10%, and a solar radiation of 600~800 W m-2;

	Beijing, China (29 March 2023): with a ambient temperature of ~30 °C, a relative humidity of 13%, and a solar radiation of ~850 W m-2;<br>Beijing, China (8 October 2023): with a ambient temperature of ~30 °C, a relative humidity of 18%, and a solar radiation of 400~600 W m-2;
Location	Ulan Buh desert, Inner Mongolia, China and Beijing, China
Access & import/export	There are no local limitations for accessing the aforementioned locations & importing/exporting our samples
Disturbance	The extra thermal radiation from surrounding buildings can affect the test results and to avoid this effect, an open and unobstructed test site is required

Reporting for specific materials, systems and methods

We require information from authors about some types of materials, experimental systems and methods used in many studies. Here, indicate whether each material, system or method listed is relevant to your study. If you are not sure if a list item applies to your research, read the appropriate section before selecting a response.

Materials & experimental systems

n/a	Involved in the study
☒	☐ Antibodies
☒	☐ Eukaryotic cell lines
☒	☐ Palaeontology and archaeology
☒	☐ Animals and other organisms
☒	☐ Clinical data
☒	☐ Dual use research of concern
☒	☐ Plants

Methods

n/a	Involved in the study
☒	☐ ChIP-seq
☒	☐ Flow cytometry
☒	☐ MRI-based neuroimaging
